# The transcription factor CAMTA2 interacts with the histone acetyltransferase GCN5 and regulates grain weight in wheat

**DOI:** 10.1093/plcell/koae261

**Published:** 2024-09-25

**Authors:** Ruijie Zhang, Kexin An, Yujiao Gao, Zhaoheng Zhang, Xiaobang Zhang, Xue Zhang, Vincenzo Rossi, Yuan Cao, Jun Xiao, Mingming Xin, Jinkun Du, Zhaorong Hu, Jie Liu, Huiru Peng, Zhongfu Ni, Qixin Sun, Yingyin Yao

**Affiliations:** Frontiers Science Center for Molecular Design Breeding, Key Laboratory of Crop Heterosis and Utilization (MOE), and Beijing Key Laboratory of Crop Genetic Improvement, China Agricultural University, Beijing 100193, China; Frontiers Science Center for Molecular Design Breeding, Key Laboratory of Crop Heterosis and Utilization (MOE), and Beijing Key Laboratory of Crop Genetic Improvement, China Agricultural University, Beijing 100193, China; Jiangsu Key Laboratory of Crop Genetics and Physiology, Yangzhou University, Yangzhou 225009, China; Frontiers Science Center for Molecular Design Breeding, Key Laboratory of Crop Heterosis and Utilization (MOE), and Beijing Key Laboratory of Crop Genetic Improvement, China Agricultural University, Beijing 100193, China; Frontiers Science Center for Molecular Design Breeding, Key Laboratory of Crop Heterosis and Utilization (MOE), and Beijing Key Laboratory of Crop Genetic Improvement, China Agricultural University, Beijing 100193, China; Frontiers Science Center for Molecular Design Breeding, Key Laboratory of Crop Heterosis and Utilization (MOE), and Beijing Key Laboratory of Crop Genetic Improvement, China Agricultural University, Beijing 100193, China; Council for Agricultural Research and Economics, Research Centre for Cereal and Industrial Crops, I-24126, Bergamo, Italy; Key Laboratory of Plant Cell and Chromosome Engineering, Institute of Genetics and Developmental Biology, Chinese Academy of Sciences, Beijing 100101, China; Key Laboratory of Plant Cell and Chromosome Engineering, Institute of Genetics and Developmental Biology, Chinese Academy of Sciences, Beijing 100101, China; Frontiers Science Center for Molecular Design Breeding, Key Laboratory of Crop Heterosis and Utilization (MOE), and Beijing Key Laboratory of Crop Genetic Improvement, China Agricultural University, Beijing 100193, China; Frontiers Science Center for Molecular Design Breeding, Key Laboratory of Crop Heterosis and Utilization (MOE), and Beijing Key Laboratory of Crop Genetic Improvement, China Agricultural University, Beijing 100193, China; Frontiers Science Center for Molecular Design Breeding, Key Laboratory of Crop Heterosis and Utilization (MOE), and Beijing Key Laboratory of Crop Genetic Improvement, China Agricultural University, Beijing 100193, China; Frontiers Science Center for Molecular Design Breeding, Key Laboratory of Crop Heterosis and Utilization (MOE), and Beijing Key Laboratory of Crop Genetic Improvement, China Agricultural University, Beijing 100193, China; Frontiers Science Center for Molecular Design Breeding, Key Laboratory of Crop Heterosis and Utilization (MOE), and Beijing Key Laboratory of Crop Genetic Improvement, China Agricultural University, Beijing 100193, China; Frontiers Science Center for Molecular Design Breeding, Key Laboratory of Crop Heterosis and Utilization (MOE), and Beijing Key Laboratory of Crop Genetic Improvement, China Agricultural University, Beijing 100193, China; Frontiers Science Center for Molecular Design Breeding, Key Laboratory of Crop Heterosis and Utilization (MOE), and Beijing Key Laboratory of Crop Genetic Improvement, China Agricultural University, Beijing 100193, China; Frontiers Science Center for Molecular Design Breeding, Key Laboratory of Crop Heterosis and Utilization (MOE), and Beijing Key Laboratory of Crop Genetic Improvement, China Agricultural University, Beijing 100193, China

## Abstract

Grain weight and size are major traits targeted in breeding to improve wheat (*Triticum aestivum* L.) yield. Here, we find that the histone acetyltransferase GENERAL CONTROL NONDEREPRESSIBLE 5 (GCN5) physically interacts with the calmodulin-binding transcription factor CAMTA2 and regulates wheat grain size and weight. *gcn5* mutant grains were smaller and contained less starch. GCN5 promoted the expression of the starch biosynthesis genes *SUCROSE SYNTHASE 2* (*Sus2*) and *STARCH-BRANCHING ENZYME Ic* (*SBEIc*) by regulating H3K9ac and H3K14ac levels in their promoters. Moreover, immunoprecipitation coupled to mass spectrometry (IP–MS) revealed that CAMTA2 physically interacts with GCN5. The CAMTA2–GCN5 complex activated *Sus2* and *SBEIc* by directly binding to their promoters and depositing H3K9ac and H3K14ac marks during wheat endosperm development. *camta2* knockout mutants exhibited similar phenotypes to *gcn5* mutants, including smaller grains that contained less starch. In *gcn5* mutants, transcripts of high-molecular-weight (HMW) *Glutenin* (*Glu*) genes were downregulated, leading to reduced HMW glutenin protein levels, gluten content, and sodium dodecyl sulfate (SDS) sedimentation volume. However, the association of GCN5 with *Glu* genes was independent of CAMTA2, since GCN5 enrichment on *Glu* promoters was unchanged in *camta2* knockouts. Finally, we identified a *CAMTA2-A^H3^* elite allele that corresponded with enhanced grain size and weight, serving as a candidate gene for breeding wheat varieties with improved grain weight.

## Teaser

A histone acetyltransferase interacts with a calmodulin-binding transcription factor and regulates wheat grain size and weight.

## Introduction

Histone acetylation involves the transfer of acetyl groups from acetyl coenzyme A (acetyl-CoA) to lysine residues on histones to eliminate lysine's positive charge. As a result, chromatin fibers are locally expanded and chromatin structure becomes loose, enabling transcription factors to bind to DNA and thus activate genes (Kumar et al. 2021). Histone acetyltransferases (HATs) are responsible for transfer of acetyl groups to specific lysine residues and thus establishing histone acetylation (Xu et al. 2023). *GENERAL CONTROL NONDEREPRESSIBLE 5* (*GCN5*) encodes a HAT that was first identified in yeast (*Saccharomyces cerevisiae*) and plays a pivotal role in epigenetic and chromatin modification in plants and animals (Georgakopoulos and Thireos 1992; Gan et al. 2021).

In Arabidopsis (*Arabidopsis thaliana*), loss of *GCN5* function results in major developmental defects, including dwarfed plants, short petals and stamens, and decreased fertility (Vlachonasios et al. 2003). In rice (*Oryza sativa*), *gcn5* mutants have fewer crown roots, have reduced primary root length, and are shorter in height than the wild type (WT) (Zhou et al. 2017). More broadly, *GCN5* is also associated with responses to biotic and abiotic stresses, including heat and salt stress, iron homeostasis, and the phosphate starvation response. Transgenic Arabidopsis and wheat (*Triticum aestivum*) lines overexpressing *GCN5* [also known as *HISTONE ACETYLTRANSFERASE 1* (*HAG1*)] have enhanced powdery mildew resistance and thermotolerance (Hu et al. 2015; Xing et al. 2015; Zheng et al. 2019; Wang et al. 2019b; Lin et al. 2022; Song et al. 2022). Moreover, *GCN5* is involved in the regulation of seed-related traits. In Arabidopsis, *GCN5* positively regulates the ratio of a-linolenic acid (ALA, C18:3) to LA (C18:2) in seed oil, and overexpression of the fatty acid desaturase family gene *FAD3* could rescue the phenotype of the *gcn5* mutant (Puttick et al. 2009; Wang et al. 2016). During maize (*Zea mays*) endosperm development, GCN5 is recruited to the promoters of zein genes, which encode storage proteins (Locatelli et al. 2009). In wheat, GCN5 interacts with the GIBBERELLIN MYB (GAMyb) transcription factor to regulate the glutenin gene *Glu*, which is associated with end-use quality (Guo et al. 2015). However, the function of GCN5 in the context of grain development remains unexplored.

GCN5 has well-established histone acetyltransferase activity and is responsible for acetylating lysine residues including H3K9, H3K14, H3K23, H3K27, and H3K36, as well as other histones such as H2 and H4 (Brownell et al. 1996; Ruiz-García et al. 1998; Benhamed et al. 2006; Gu et al. 2022). Arabidopsis GCN5 regulates H3K14/K9 acetylation levels of *CAPRICE* (*CPC*) and *GLABROUS1* (*GL1*) genes for trichome initiation and for iron homeostasis by mediating H3K9ac and H3K14ac levels at *FERRIC REDUCTASE DEFECTIVE3* (*FRD3*) (Xing et al. 2015; Wang et al. 2019a). In wheat, GCN5 activates *PHYTOALEXIN DEFICIENT* (*PAD4*) expression by increasing the levels of H3K9ac and H3K14ac in response to powdery mildew challenge (Song et al. 2022).

GCN5 forms an independent HAT module by interacting with Alteration/Deficiency in Activation 2 (ADA2), alteration/deficiency in activation-3 (ADA3), and SAGA complex-associated factor 29 (SGF29) (Espinola-Lopez and Tan 2021). In plants, the HAT module, structural core (TAF and SPT modules), splicing modules, and plant-specific subunits SCS1, SCS2A, and SCS2B form the SAGA (Spt–Ada–Gcn5 acetyltransferase) complex involved in the transcriptional activation of a series of genes that participates in plant growth, development, and stress responses (Grant et al. 1997; Hampsey 1997; Weake et al. 2011; Kong et al. 2017; Zhou et al. 2017; Li et al. 2019; Wu et al. 2021). Two genes encode ADA2 homologs ADA2a and ADA2b in plants and animals (Stockinger et al. 2001; Muratoglu et al. 2003; Gamper et al. 2009). Both homologs interact with GCN5 to enhance its transferase activity in vitro and act as a molecular bridge connecting HATs and other components of the SAGA complex (Mao et al. 2006; Lee et al. 2011).

GCN5 regulates histone acetylation in the promoters of target genes recruited by transcription factors. For example, *Populus trichocarpa* GCN5 interacts with AREB1-2 (ABRE binding protein) to enhance H3K9ac acetylation of *NAC* [NAM (No Apical Meristem)–ATAF (Arabidopsis Transcription Activation Factor)–CUC (Cup-shaped Cotyledons)] genes in drought tolerance (Li et al. 2019). In wheat, GCN5 interacts with the transcription factor PLATZ5 (a plant-specific zinc-binding protein) to activate *PAD4* expression and is involved in powdery mildew resistance (Song et al. 2022). GCN5 also interacts with transcription factors NACL and HsfA1 (a heat-shock transcription factor) to promote heat tolerance in wheat (Lin et al. 2022; Wang et al. 2023). However, the identity of components of the GCN5 complex and its mechanisms in regulating gene expression during wheat seed development remain unknown.

CAMTAs (calmodulin-binding transcription activators) can activate or repress gene expression to affect plant growth and development (Bouché et al. 2002; Choi et al. 2005; Yue et al. 2015; Yang et al. 2020). CAMTAs contain a CG-1 DNA-binding domain specifically recognizing the (A/C/G)CGCG(G/T/C) motif (CGCG box), a TIG domain taking part in nonspecific DNA contacts, several ANK (ankyrin repeats) domains responsible for protein–protein interactions, an IQ motif, and a CaMB (calmodulin-binding domain) known to bind CaM proteins (Sedgwick and Smerdon 1999; Bähler and Rhoads 2002; Yang and Poovaiah 2002; Finkler et al. 2007). Overexpression of *CAMTA12* enhances soybean (*Glycine max*) drought tolerance (Noman et al. 2019). Arabidopsis CAMTA3/SR1 acts as a repressor to negatively regulate plant immunity by downregulating the expression of *SYSTEMIC ACQUIRED RESISTANCE DEFICIENT 1* (*SARD1*) *and CALMODULIN-BINDING PROTEIN 60g* (*CBP60g*) (Du et al. 2009; Yuan et al. 2018a; Sun et al. 2020). By contrast, CAMTA3/SR1 also acts as a transcriptional activator for *C-REPEAT-BINDING FACTOR DEHYDRATION-RESPONSIVE ELEMENT-BINDING FACTOR 1* (*CCBF2/DREB1C*) expression in the cold-stress response (Doherty et al. 2009; Yuan et al. 2018b). In addition, *CAMTAs* are involved in the regulation of fruit development and ripening in tomato (*Solanum lycopersicum*) (Yang et al. 2012). In wheat, *CAMTA4* negatively regulates of the defense response against the fungal pathogen *Puccinia triticina* (Wang et al. 2019c). Although CAMTAs are annotated in wheat (Yang et al. 2020), there are only few reports of their function.

Here, we find that GCN5 regulates starch and seed storage protein (SSP) accumulation during wheat grain development and proposes a molecular mechanism of GCN5 function. We show that CAMTA2 physically interacts with GCN5 to regulate grain size, weight, and starch accumulation. CAMTA2 activates starch biosynthesis by directly binding to the CGCG motif in the *Sus2* and *SBEIc* promoters by interacting with GCN5 to establish H3K9ac and H3K14ac histone marks during endosperm development. CAMTA2 binds to the promoters of genes related to starch metabolism and *Glu* genes, and although the RNA levels of genes related to both starch metabolism and *Glu* genes are reduced in field-grown *camta2* knockouts, only starch content is reduced, and contents of SSPs are unchanged compared to WT, indicating the principal function of CAMTA2 in wheat endosperm may be related to regulation of starch accumulation. We identified an elite allele of *CAMTA2-A^H3^* that positively corresponds with grain size and weight. Our study expands the role of histone acetylation in the regulation of grain weight.

## Results

### Downregulation of *GCN5* leads to smaller kernels and decreased starch content

We previously generated a *GCN5* mutant using CRISPR–Cas9 by targeting a conserved region in the 3 *GCN5* homoeologs in the c.v. Fielder background that contained homozygous mutations in the A and B homoeologs only, with the D homoeolog unaffected (Zheng et al. 2021). Here, we obtained 3 additional genetically independent homozygous mutants including *gcn5-AABBdd*, *gcn5-AAbbdd*, and *gcn5-aaBBdd* and 2 heterozygous lines including *gcn5-Aabbdd* and *gcn5-aaBbdd.* All of these mutant genotypes were fertile. We also recovered lines with mutations in all 3 *GCN5* homoeologs expressing undetectable levels of GCN5 protein (Supplementary Fig. S1A), but these lines were all sterile and therefore excluded from further analysis.

**Figure 1.**
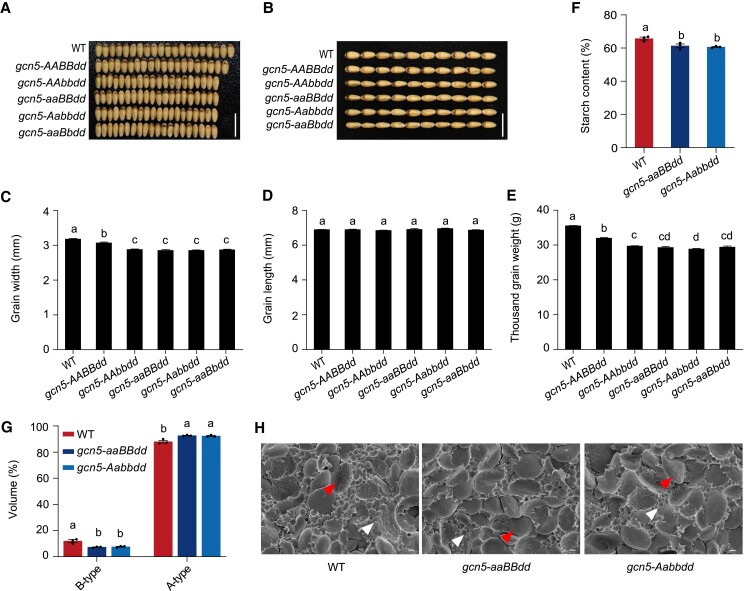
Grain phenotypes of wheat *gcn5* mutants under field conditions. Grain width **A, C)**, grain length **B, D)**, and thousand-grain weight **E)** of the WT c.v. Fielder and *gcn5* mutants. Scale bars = 1 cm **A, B)**. For **C, D** and **E)**, data are the mean ± SD of *n* = 8 replicates comprising > 300 grains per replicate. **F)** Starch content in WT and *gcn5* mutant seeds. Data are the mean ± SD of *n* = 3 replicates. **G)** Relative starch granule size distribution in WT and *gcn5* mutants. The proportion of large A-type starch granules ranging from 10 to 35 *µ*m in diameter and small B-type starch granules ranging from 1 to 10 *µ*m in diameter was quantified. Data are the mean ± SD of *n* = 3 replicates. **H)** Representative scanning electron-microscopy images of starch granules from the mature seeds of WT and *gcn5* mutants. The arrowheads indicate A-type (10 to 35 *μ*m) and B-type (1 to 10 *μ*m) starch granules, respectively. Scale bars = 50 *μ*m. For **C** to **G)**, the effect of genotype on statistically significant differences in means for y-axis traits was determined by one-way ANOVA. Statistically significant differences between means are depicted by different letters (*P* < 0.05).

Under field conditions, viable homozygous *gcn5* mutant lines with homozygous loss-of-function mutations in 1 or 2 of the 3 *GCN5* homoeologs expressed less GCN5 protein than WT c.v. Fielder (Supplementary Fig. S1B), had smaller kernels, narrower grain width, and smaller thousand-grain weight, while grain length was unchanged compared to WT (Fig. 1, A to E). No visible phenotypic differences were detected in *gcn5* mutant mature plants relative to WT, including plant height, spike length, spikelet number, and grain number per spike (Supplementary Fig. S2, A to E). *gcn5* mutant lines with two or more homoeologs knocked out (namely, *gcn5-AAbbdd*, *gcn5-aaBBdd*, *gcn5-Aabbdd*, and *gcn5-aaBbdd*) had smaller kernels compared to *gcn5-AABBdd* containing 1 homoeolog mutated (Fig. 1, A, C, and E).

*gcn5-aaBBdd* and *gcn5-Aabbdd*, with more pronounced seed defect phenotypes, were selected for further analysis to determine whether starch contents and starch granule size are disturbed in *gcn5* mutants with smaller kernels. B-type starch granules initiate between 12 and 16 d after pollination (DAP) and continue to form in the later stages of wheat grain development (Langeveld et al. 2000). Here, starch contents in *gcn5-aaBBdd* and *gcn5-Aabbdd* were statistically significantly decreased compared to WT (Fig. 1F). The proportion of large A-type (∼20 *µ*m) and small B-type (∼5 *µ*m) starch granules was higher and lower in *gcn5* lines, respectively, compared to WT (Fig. 1, G and H). We measured the cell size and cell number of 15 DAP endosperm from *gcn5-aaBBdd*, *gcn5-Aabbdd*, and WT, and the results indicate that there is no significant alteration in either cell size or cell number between the *gcn5* mutants and WT (Supplementary Fig. S2, F to H). These results indicated that *GCN5* affects starch biosynthesis and the ratio of A- and B-type starch granules in wheat.

### GCN5 associates with loci involved in starch biosynthesis by mediating histone acetylation

**Figure 2.**
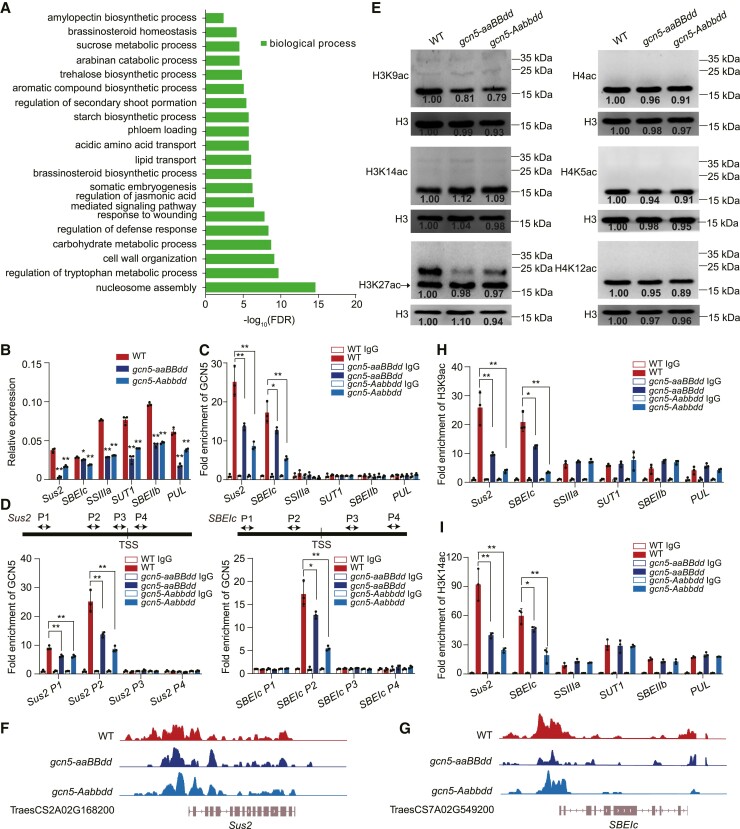
Genes and biological processes regulated by GCN5 in wheat. **A)** GO analysis of genes downregulated in 25 DAP endosperm of *gcn5* mutants compared to WT. Only genes differentially expressed in both mutants were considered. *P*-values were adjusted by the Benjamini–Hochberg correction and only statistically significant GO categories (FDR < 0.05) are shown. **B)** RT–qPCR of *Sus2* (sucrose synthase), *SSIIIa* (starch synthase IIIa), *SUTI* (sucrose transporter), *SBEIc* (starch-branching enzyme Ic), *SBEIIb* (starch-branching enzyme IIb), and *PUL* (limit dextrinase-type starch-debranching enzyme) in WT and *gcn5* endosperm at 25 DAP. Relative expression was normalized to *ACTIN*. Data are the mean ± SD of *n* = 3 replicates. Statistically significant differences between means of genotypes were determined by Student's *t* test against the WT control and are indicated by *, *P* < 0.05, and **, *P* < 0.01. **C)** ChIP–qPCR of GCN5 enrichment at loci related to starch biosynthesis that are downregulated in *gcn5*. Data are the mean ± SD of *n* = 3 replicates. Statistically significant differences are indicated by *, *P* < 0.05, and **, *P* < 0.01, as determined by Student's *t* test. **D)** The positions of the primer sets (arrows) used in the ChIP assay relative to the transcriptional start sites (TSSs) are shown (*top*). ChIP–qPCR (*bottom*) of GCN5 enrichment at *Sus2* and *SBEIc* in *gcn5*. Data are the mean ± SD of *n* = 3 replicates. Statistically significant differences are indicated by *, *P* < 0.05, and **, *P* < 0.01, as determined by Student's *t* test. **E)** Immunoblot of H3K9ac, H3K14ac, H3K27ac, H4ac, H4K5ac, and H4K12ac marks in 20 DAP endosperm from WT and *gcn5* mutants. Black arrowhead indicates H3K27ac. Nuclear proteins were loaded onto 10% (*w*/*v*) SDS–PAGE gels and H3 was used as a loading control. **F, G)** Genome browser views of H3K9ac marks at *Sus2*  **F)** and *SBEIc*  **G)** in WT and *gcn5*. **H, I)** H3K9ac accumulation **H)** and H3K14ac accumulation **I)** in genes related to starch biosynthesis downregulated in *gcn5*. ChIP–qPCR was performed and data are the mean ± SD of *n* = 3 replicates. Statistically significant differences are indicated by *, *P* < 0.05, and **, *P* < 0.01, as determined by Student's *t* test.

To identify genes bound by GCN5, we performed RNA-seq analysis using RNA isolated from 25 DAP endosperm from WT, *gcn5-aaBBdd*, and *gcn5-Aabbdd* mutants. Genes differentially expressed simultaneously in both mutants were analyzed. A total of 1,607 genes were downregulated, and 832 genes were upregulated in the mutants compared to WT (Supplementary Data Set 1). In the *gcn5-aaBBdd* mutant, there was a statistically significant reduction in *GCN5-A* and *GCN5-D* expression, while *GCN5-B* remained unaltered. In the *gcn5-Aabbdd* mutant, *GCN5-B* and *GCN5-D* were statistically significantly decreased, with no change detected for *GCN5-A* (Supplementary Fig. S1C). Gene Ontology (GO) analysis revealed that genes downregulated in *gcn5* are enriched for regulation of tryptophan metabolic process, carbohydrate metabolic process, brassinosteroid (BR) biosynthetic process, starch biosynthetic process, sucrose metabolic process, and amylopectin biosynthetic process (Fig. 2A). Genes upregulated in *gcn5* were enriched in GO terms related to amino acid transmembrane transport, terpene biosynthetic process, and killing of cells of other organism (Supplementary Fig. S3A). Reduced levels of BR lead to smaller grains and lower grain weight in rice and wheat (Hong et al. 2002; Xiong et al. 2022; Lyu et al. 2024; Wei and Jiao 2024). Two genes involved in BR biosynthesis were confirmed as downregulated in *gcn5* mutants by RT–qPCR (Supplementary Fig. S3B), namely, TraesCS4A02G078000, which encodes a cytochrome P450 90B2 and is a putative ortholog of rice *DWARF4*/*CYP90B2*, and TraesCS2A02G013500, a putative ortholog of rice *brd1*/*DWARF* (Hong et al. 2002; Mori et al. 2002; Sakamoto et al. 2006). This suggests that GCN5 may regulate grain size and weight through modulation of the BR biosynthetic pathway. In agreement with the reduced starch levels in *gcn5* mutants, 21 genes related to starch metabolism were downregulated (Supplementary Fig. S4 and Supplementary Data Set 2). Downregulation of 6 genes was confirmed by RT–qPCR, including *Sus2* (encoding a sucrose synthase), *SBEIc* (encoding starch-branching enzyme Ic), *SSIIIa* (encoding starch synthase IIIa), *SUT1* (encoding a sucrose transporter), *SBEIIb* (encoding starch-branching enzyme IIb), and *PUL* (encoding limit dextrinase-type starch-debranching enzyme) (Fig. 2B). *NF-YB1* encodes a transcription activator controlling starch biosynthesis (Liu et al. 2023) and was downregulated in *gcn5* mutants, indicating that GCN5 might also regulate starch accumulation by modulating *NF-YB1* expression (Supplementary Fig. S3C).

To determine the genes directly targeted by GCN5, ChIP was performed using an antibody against native GCN5 protein using chromatin samples from 20 DAP endosperm isolated from WT, *gcn5-aaBBdd*, and *gcn5-Aabbdd*. We focused on 6 genes related to sucrose and starch metabolism that are differentially expressed in *gcn5.* ChIP–qPCR results indicated that the enrichment of GCN5 in the *Sus2* and *SBEIc* promoters was reduced in *gcn5-aaBBdd* and *gcn5-Aabbdd* backgrounds and was the same as WT levels at the promoters of the other 4 assayed genes (Fig. 2C). We designed an additional 3 pairs of primers covering promoter and gene bodies of *Sus2* and *SBEIc*, and the result showed that GCN5 is enriched in the promoter region but not in gene bodies (Fig. 2D). The results suggested that GCN5 associates directly with *Sus2* and *SBEIc*. To confirm specific binding of GCN5 to its targets, we randomly selected 15 genes evenly distributed on wheat chromosomes and only 1 gene was observed in GCN5 enrichment (Supplementary Fig. S3D), suggesting the specific binding of GCN5 to its targets. GCN5 has histone acetyltransferase activity and is responsible for histone acetylation of chromatin and transcriptional regulation (Gan et al. 2021). Among known histone acetylation modifications at various lysine residues, the level of histone H3 acetylation at lysine 9 (H3K9ac) was decreased in *gcn5-aaBBdd* and *gcn5-Aabbdd* mutant lines compared with WT (Fig. 2E). Next, we used a Cleavage Under Targets and Tagmentation (CUT&Tag) assay with an anti-H3K9ac antibody to assay loci with deceased H3K9ac marks in *gcn5-aaBBdd* and *gcn5-Aabbdd* mutant lines. The H3K9ac levels of 761 genes were downregulated in *gcn5* compared to WT (Supplementary Data Set 3). Among GCN5 target genes related to starch biosynthesis, promoters of *Sus2* and *SBEIc* have lower H3K9ac levels in *gcn5-aaBBdd* and *gcn5-Aabbdd* compared to WT (Fig. 2, F and G), which was confirmed by ChIP–qPCR (Fig. 2H). Additionally, GCN5 is well known for H3K9 and H3K14 acetylation in wheat (Song et al. 2022). We further performed ChIP–qPCR with an anti-H3K14ac antibody and found that H3K14ac enrichment in the promoters of *Sus2* and *SBEIc* was reduced in *gcn5* mutants (Fig. 2I). These results collectively indicate that the downregulation of *Sus2* and *SBEIc* expression is associated with the decreased levels of H3K9ac and H3K14ac in their promoters.

### The transcription factor CAMTA2 physically interacts with GNC5

**Figure 3.**
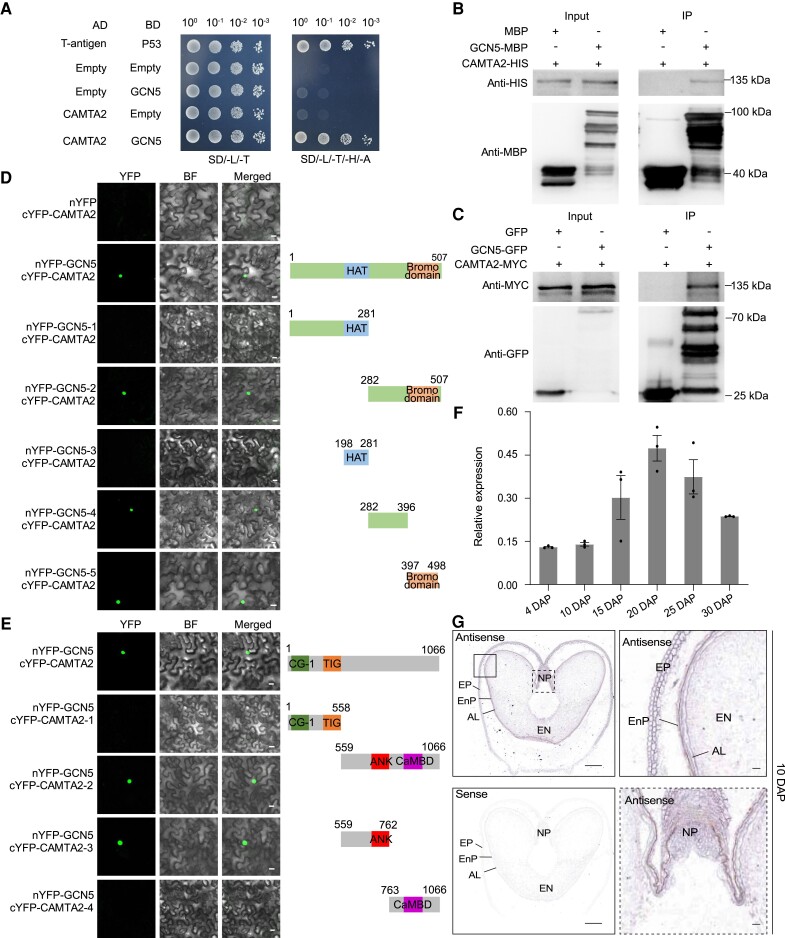
GCN5 physically interacts with the transcription factor CAMTA2. **A)** Representative images of Y2H assay showing interaction of GCN5 and CAMTA2. *GCN5* was cloned into the pGBKT7 vector and *CAMTA2* was cloned into the pGADT7 vector. Combinations of the T-antigen and P53 were used as a positive control. **B)**  *In vitro* pull-down assay showing interaction of GCN5–MBP and CAMTA2–6xHis fusion protein. CAMTA2–6xHis protein was incubated with immobilized MBP or GCN5–MBP proteins. “+” and “−” indicate the presence and absence of the indicated proteins. Proteins were detected by immunoblotting with anti-MBP and anti-HIS antibodies. **C)** Co-IP assay indicating GCN5 interacts with CAMTA2. CAMTA2–MYC was co-infiltrated into *N. benthamiana* leaves with GFP or GCN5–GFP. “+” and “−” indicate the presence and absence of the indicated proteins. Proteins were detected by immunoblotting with anti-GFP and anti-MYC antibodies. **D)** BiFC and schematics of *GCN5* deletion constructs. *GCN5* was cloned in frame with the N–YFP vector with the respective deletions and *CAMTA2* was cloned in frame with the C–YFP vector. BF, bright field. Scale bars = 20 *μ*m. **E)** BiFC and schematics of *CAMTA2* deletion constructs. *GCN5* was cloned in frame with the N–YFP vector and *CAMTA2* was cloned in frame with the C–YFP vector with the respective deletions. BF: Bright field. Scale bars = 20 *μ*m. **F)** RT–qPCR of *CAMTA2* in whole seed (4 DAP) and developing endosperm (10 to 30 DAP). Relative expression was normalized to *ACTIN*. Data are the mean ± SD of *n* = 3 replicates. **G)** Representative images of in situ hybridization assays with 10 DAP transverse seed sections hybridized with antisense and sense *CAMTA2* probes. EP, exocarp; EnP, endocarp; AL, aleurone layer; NP, nucellar projection; En, endosperm. Scale bars = 500 *μ*m.

To identify protein complexes that GCN5 is a member of, we used immunoprecipitation coupled to mass spectrometry (IP–MS) with an anti-GCN5 antibody in samples isolated from WT c.v. Fielder and identified 62 proteins that interact with GCN5 in two independent biological replicates (Supplementary Data Set 4). Among the interacting proteins identified, TraesCS4A02G407100, annotated as the calmodulin-binding transcription factor CAMTA2 (Yang et al. 2020), had a high IP–MS score and implies a potential role in transcriptional regulation. When transiently expressed in *Nicotiana benthamiana* leaves, CAMTA2–GFP localized to the nucleus, with GCN5–GFP and the organelle marker SPL16–RFP (Supplementary Fig. S5A). We verified the physical interaction between GCN5 and CAMTA2 using 4 independent approaches including yeast two-hybrid (Y2H) assay (Fig. 3A), in vitro pull-down assay (Fig. 3B) and in vivo co-immunoprecipitation (Co-IP) assay in transiently transgenic *N. benthamiana* leaves (Fig. 3C). Bimolecular fluorescence complementation (BiFC) indicated the GCN5–CAMTA2 interaction can occur, at least in transiently transgenic *N. benthamiana* leaves (Fig. 3D, Supplementary Fig. S5B). Amino acids 282 to 498 of GCN5 (including the bromo domain) are sufficient for interaction with the ANK domain of CAMTA2 (Fig. 3, D and E), which is responsible for protein–protein interactions (Rubtsov and Lopina 2000; Song et al. 2006).

Toward placing the GCN5–CAMTA2 physical interaction in a spatial gene expression context in planta, we queried publicly available wheat gene expression atlas data (Ramírez-González et al. 2018) (Supplementary Fig. S6). The 3 *CAMTA2* homoeologs are highly expressed in the endosperm, with transcripts detectable during early kernel development and plateauing at 20 and 25 DAP before, then decreasing (Fig. 3F). RNA in situ hybridization showed *CAMTA2* homoeologs were expressed in the exocarp, endocarp, nucellar projection, aleurone layer, and endosperm of 10 and 20 DAP seeds (Fig. 3G and Supplementary Fig. S5C). These observations collectively implicate *CAMTA2* in seed development.

### Knockout of *CAMTA2* leads to small kernels and less starch

**Figure 4.**
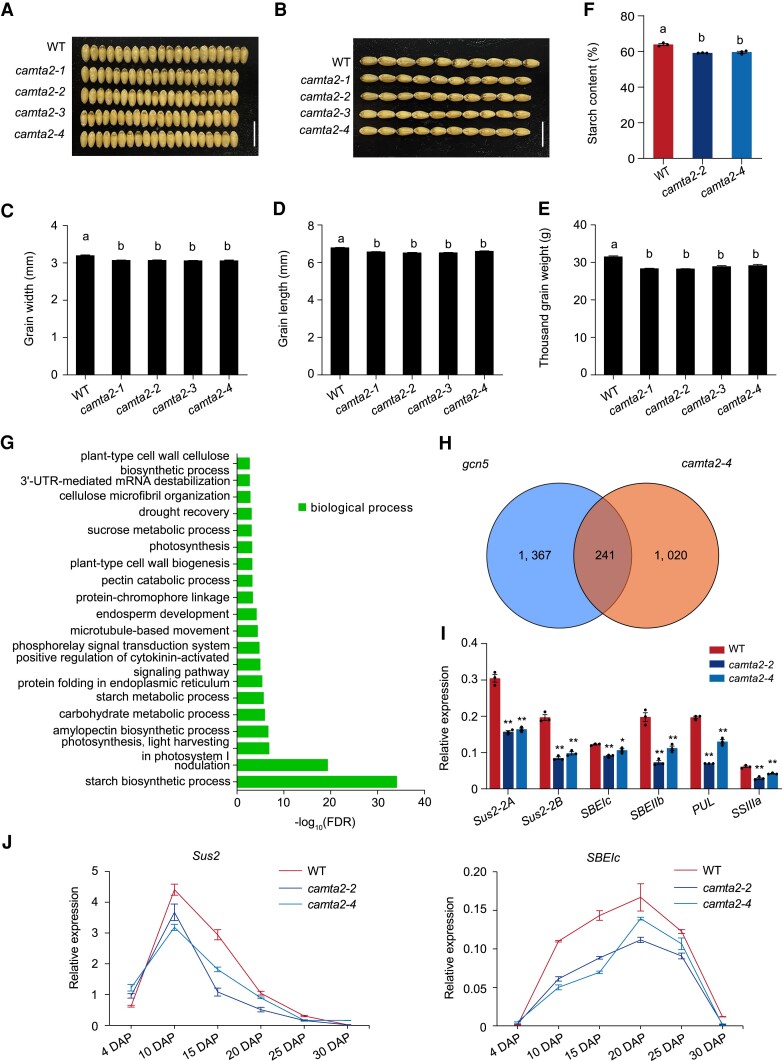
Characterization of *camta2* mutant phenotypes. **A** to **E)** Grain width **A, C)**, grain length **B, D)**, and thousand-grain weight **E)** in WT and *camta2* mutants. Scale bars = 1 cm for **A** and **B)**. For **C, D,** and **E)** data are the mean ± SD and eight replicates were used with > 300 grains per replicate. **F)** Starch contents in WT and *camta2* mutants. Data are the mean ± SD of *n* = 3 replicates. For **C** to **F)**, the effect of genotype on statistically significant differences in means for y-axis traits was determined by one-way ANOVA. Statistically significant differences between means are depicted by different letters (*P* < 0.05). **G)** GO analysis of genes downregulated in RNA samples isolated from 25 DAP *camta2*–*4* endosperm compared to WT. *P*-values were adjusted by the Benjamini–Hochberg correction, and only statistically significant GO categories (FDR < 0.05) are shown. **H)** Venn diagram of overlap between genes downregulated in *gcn5* mutants and downregulated genes in *camta2* mutants compared with WT. **I)** RT–qPCR of *Sus2-2A* and *Sus2-2B* (sucrose synthases), *SBEIc* (starch-branching enzyme Ic), *SBEIIb* (starch-branching enzyme IIb), *PUL* (limit dextrinase-type starch-debranching enzyme), and *SSIIIa* (starch-synthase IIIa) in WT, *camta2*–*2*, and *camta2*–*4* endosperm at 25 DAP. Relative expression was normalized to *ACTIN*. Data are the mean ± SD of *n* = 3 replicates. Statistically significant differences are indicated by *, *P* < 0.05, and **, *P* < 0.01, as determined by Student's *t* test. **J)** Expression of *Sus2* (sucrose synthase) and *SBEIc* (starch-branching enzyme Ic) during seed development in WT, *camta2*–*2*, and *camta2*–*4*. Relative expression was normalized to *ACTIN*. Data are the mean ± SD of *n* = 3 replicates.

Using a sgRNA targeting a conserved region in exon 1 of all 3 *CAMTA2* homoeologs (Supplementary Fig. S7A), 4 genetically independent mutant lines (designated *camta2*-*1*, *camta2*-*2*, *camta2*-*3*, and *camta2*-*4*) were obtained by CRISPR–Cas9 gene editing that express undetectable CAMTA2. No off-target editing events were apparent by PCR analysis (Supplementary Fig. S7, B and C). These knockout mutants have similar phenotypes to *gcn5* mutants, including decreased grain width (Fig. 4, A and C), decreased grain length (Fig. 4, B and D) and a smaller thousand-grain weight (Fig. 4E). Starch contents in *camta2*-*2* and *camta2*-*4* were statistically significantly decreased compared to WT (Fig. 4F). Cell number and cell size in 15 DAP *camta2* endosperm had no obvious change versus WT (Supplementary Fig. S7, D to F). To explore downstream targets of *CAMTA2*, we performed RNA-seq using RNA isolated from the endosperm of WT and *camta2*-*4* at 25 DAP. Owing to the similar phenotypes of *camta2*-*4* and *gcn5* mutants, we focused on the 1,261 genes that were downregulated in *camta2*-*4* versus WT (Supplementary Data Set 5), which were enriched in starch biosynthetic process, amylopectin biosynthetic process, starch metabolic process, and sucrose metabolic process (Fig. 4G). Among them, 241 genes were also downregulated in the *gcn5* mutants compared to WT (Fig. 4H) and included 17 genes related to sucrose and starch metabolism (Supplementary Fig. S8A and Supplementary Data Set 6). Among these 17 genes, 6 genes including *Sus2-2A*, *Sus2-2B*, *SBEIc*, *SBEIIb*, *PUL*, and *SSIIIa* were confirmed by RT–qPCR in *camta2*-*2* and *camta2*-*4* backgrounds (Fig. 4I). *Sus2* and *SBEIc* decreased in *camta2* at different stages of seed development (Fig. 4J). These results suggested the co-regulation of *GCN5* and *CAMTA2* in the context of starch accumulation. Moreover, *PBF/LYS3*, a starch accumulation activator (Orman-Ligeza et al. 2020), was also downregulated in *camta2* mutants (Supplementary Fig. S8B), suggesting that CAMTA2 might modulate starch biosynthesis genes by regulating PBF/LYS.

### CAMTA2 activates *Sus2* and *SBEIc* expression by recruiting GCN5

**Figure 5.**
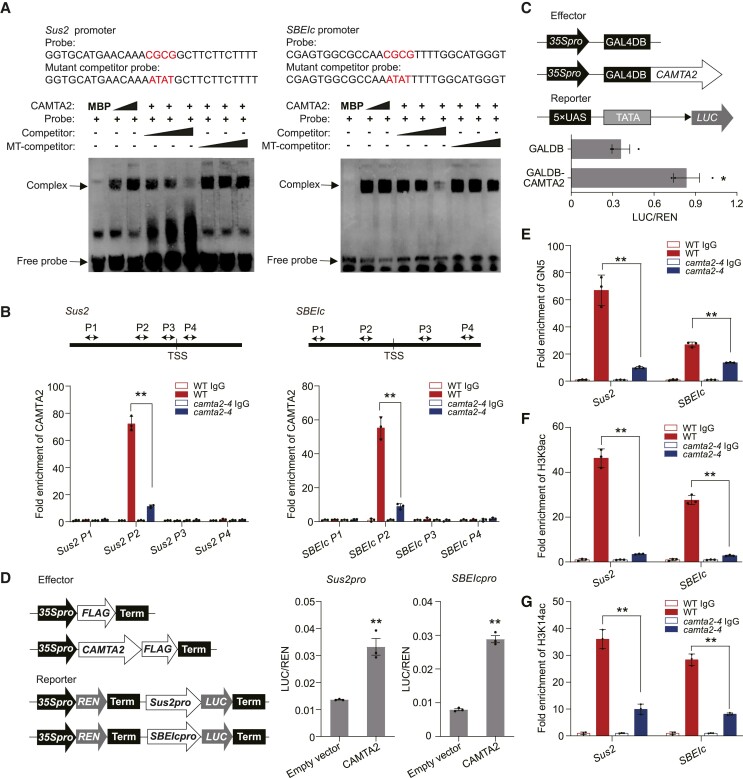
CAMTA2 binds to the *Sus2* and *SBEIc* promoters and acts as a transcriptional activator by recruiting GCN5. **A)** Representative results of EMSA with recombinant CAMTA2 DNA-binding domain (amino acids 1–200) fused to MBP, with 2 biotin-labeled probes, unlabeled competitor probes, and mutant competitor probes (MT competitor) derived from the promoters of *Sus2* and *SBEIc*, respectively. The sequences of the probes and mutant competitors are shown. “+”, “–” and triangles indicate the presence, absence and increasing concentrations of the indicated probes, proteins or competitors. **B)** The positions of the primer sets (arrows) used in the ChIP assay relative to the transcriptional start sites (TSSs) are shown (*top*). ChIP–qPCR (*bottom*) of CAMTA2 enrichment at *Sus2* and *SBEIc* in *camta2*. Data are the mean ± SD of *n* = 3 replicates. Statistically significant differences are indicated by **, *P* < 0.01, as determined by Student's *t* test. **C)** Schematic of constructs (*top*) and quantification (*bottom*) of transcription activation assays in wheat leaf protoplasts. The internal control Renilla luciferase (REN) was driven by the CaMV *35S* promoter and REN activity was normalized to firefly luciferase (LUC) activity from the reporters of interest. Data are the mean ± SD of *n* = 3 replicates reporting the LUC:REN ratio. Statistically significant differences between means are indicated by *, *P* < 0.05, as determined by Student's *t* test. **D)** Dual-luciferase reporter assay assessing the ability of CAMTA2–FLAG to activate promoters of interest. Schematics of the effector (*top* left) and reporter (*bottom* left) T-DNA regions. For the center and right panels, y-axes indicate the ratio of LUC:REN activity. Data are the mean ± SD of *n* = 3 replicates reporting the LUC:REN ratio. Statistically significant differences between means are indicated by **, *P* < 0.01, as determined by Student's *t* test. **E** to **G)** ChIP–qPCR assay of relative GCN5 enrichment **E)**, H3K9ac enrichment **F)** and H3K14ac enrichment **G)** in *Sus2* and *SBEIc* promoters in WT versus *camta2-4*. Data are the mean ± SD of *n* = 3 replicates. Statistically significant differences between means are indicated by **, *P* < 0.01, as determined by Student's *t* test.

CAMTA2 carries a predicted CG-1 DNA-binding domain (residues 22 to 134) at its N-terminus, which specifically recognizes (A/C/G) CGCG (G/T/C) motifs (Yang and Poovaiah 2002). We saw that among the 17 downregulated genes involved in sucrose and starch metabolism in both *gcn5* and *camta2* mutants compared with WT, 16 of these contain CGCG motifs in their promoter regions (Supplementary Data Set 6). To confirm the DNA-binding domain activity of CAMTA2, electrophoretic mobility shift assay (EMSA) indicated that amino acids 1 to 200 of CAMTA2 can bind to the CGCG motif present in the promoter of *Sus2* instead of predicted CG-1 DNA-binding domain (residues 22 to 134; Supplementary Fig. S9A). CAMTA2–MBP (1 to 200 aa) was selected for further analysis and binds to the CGCG motif in the promoters of *Sus2* and *SBEIc* (Fig. 5A). ChIP–qPCR using an antibody against CAMTA2 confirmed that CAMTA2 was enriched in promoter region around its binding motif but not in gene bodies of *Sus2* and *SBEIc* in endosperm (Fig. 5B). To confirm specific binding of CAMTA2 to its targets, we randomly selected 13 genes evenly distributed on wheat chromosomes, and no gene was observed as having detectable CAMTA2 enrichment (Supplementary Fig. S9B), suggesting the specific binding of CAMTA2 to its targets. To test the regulatory effect of CAMTA2 on the *Sus2* and *SBEIc* promoters, reporter assays showed that CAMTA2–FLAG acts as an activator of the *Sus2* and *SBEIc* promoters when transiently expressed in wheat leaf protoplasts (Fig. 5, C and D). These data suggested that CAMTA2 can bind to and directly activate the transcription of *Sus2* and *SBEIc*.

Given that CAMTA2 and GCN5 physically interact to target the *Sus2* and *SBEIc* promoter regions, we hypothesized that GCN5 is recruited to target genes by the transcription factor CAMTA2. ChIP–qPCR in WT and *camta2*-*4* endosperm at 20 DAP indicated GCN5 enrichment in the *Sus2* and *SBEIc* promoters was reduced in *camta2*-*4* (Fig. 5E). Moreover, H3K9ac and H3K14ac levels in the promoters of *Sus2* and *SBEIc* were also substantially lower in *camta2*-*4* compared to WT (Fig. 5, F and G). Overall, these findings suggested that CAMTA2 can recruit GCN5 to regulate *Sus2* and *SBEIc* expression during wheat seed development.

### *CAMTA2-A* haplotypes positively correlate with grain size and weight

**Figure 6.**
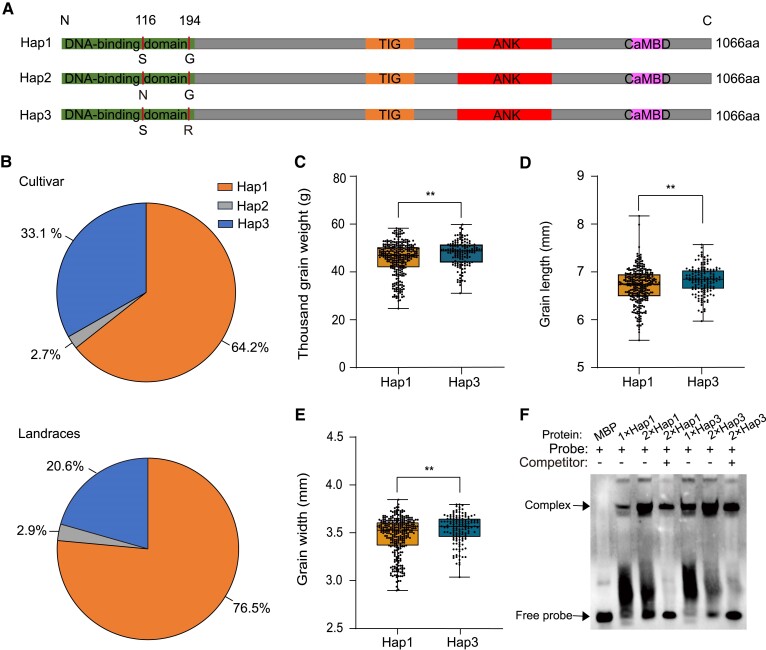
The *CAMTA2-A* haplotype 3 is associated with larger and heavier grain. **A)** Schematic of protein products encoded by *CAMTA2-A* haplotypes (*CAMTA2-A^H1–3^*) with the indicated polymorphisms. **B)** Distribution of *CAMTA2-A* haplotypes (depicted in A) among 377 modern cultivars and 68 landraces. **C** to **E)** Thousand-grain weight, grain length, and grain width in *CAMTA2-A* haplotypes. *n* = 294 accessions for Hap1 and *n* = 139 for Hap3. The effect of genotype on statistically significant differences in means for y-axis traits are indicated by **, *P* < 0.01, as determined by Student's *t* test. In the boxplots, the middle line is plotted at the median, the box is from the 25th to 75th percentiles, and the whiskers represent the minimum and the maximum value and display all the data points of the samples. **F)** EMSA of the recombinant CAMTA2 DNA-binding domain from Hap1 and Hap3 fused to MBP. Biotin-labeled probes and unlabeled competitor probes were derived from the promoter of *Sus2*. 4 *µ*g (1×), 8 *µ*g (2×) of Hap1and Hap3 protein fused to MBP was loaded onto gels for EMSA.

We used single-nucleotide polymorphism (SNP) analysis to evaluate diversity in *CAMTA2* homoeologs in a wheat germplasm collection of 445 accessions comprising 377 modern cultivars and 68 landraces (Wang et al. 2021). No polymorphisms were found in the coding sequence of *CAMTA2-B* and *CAMTA2-D* homoeologs. Three haplotypes (Hap1–3) were identified for *CAMTA2-A* and were assigned *CAMTA2-A^H1-3^*, which differ at nucleotide positions G347A and G580A relative to the translational start site, leading to amino acid substitutions at positions 116 and 194 (Fig. 6A). We developed a kompetitive allele-specific PCR (KASP) marker to distinguish Hap2 from the other 2 haplotypes (Supplementary Fig. S10A) and another KASP marker to distinguish Hap3 from the other 2 haplotypes (Supplementary Fig. S10B). We used these 2 KASP markers to genotype the germplasm (Supplementary Data Set 7). Two hundred and ninety-four, 12, and 139 accessions each harbor Hap1, Hap2, and Hap3, respectively (Supplementary Data Set 7). Hap1 was most abundant and Hap2 was the least abundant in both modern cultivars and landraces (Fig. 6B).

Considering the low percentage of Hap2, we focused on comparing the association between Hap1 and Hap3 with grain-related traits. Accessions containing Hap3 have large kernels with longer and wider grains and higher thousand-grain weight (Fig. 6, C to E). Thousand-grain weight for accessions with Hap3 (47.49 g ± 5.26) was statistically significantly higher than those with Hap1 (45.22 g ± 6.94). Interestingly, percentage of Hap3 in modern cultivars (33.1%) was higher than that in landraces (20.6%). EMSA indicated both CAMTA2-A^H1^ and CAMTA2-A^H3^ can bind the *Sus2* promoter, with Hap3 showing a stronger binding affinity to the *Sus2* promoter over CAMTA2-A^H1^ (Fig. 6F). Overall, these results indicate that *CAMTA2-A^H3^* represents an elite allele associated with larger and heavier grain and underscores its suitability for use in breeding programs.

### CAMTA2 and GCN5 might function independently in SSP accumulation and starch accumulation

**Figure 7.**
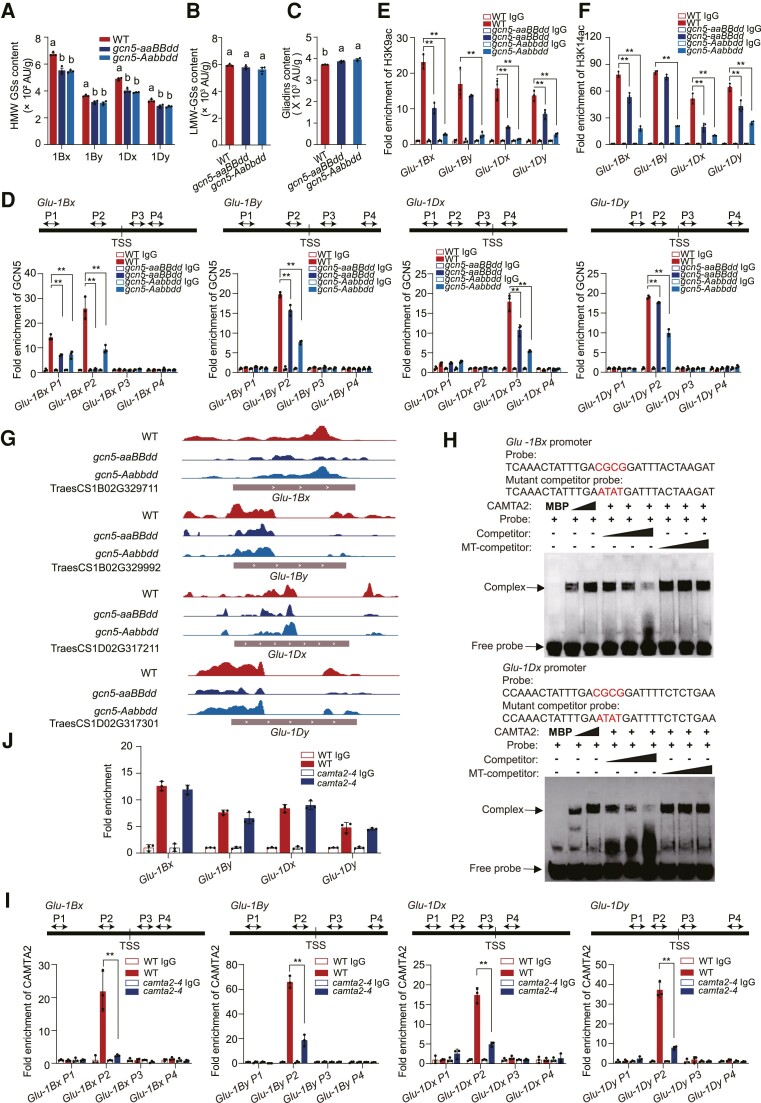
CAMTA2 and GCN5 might function independently in SSP accumulation and starch accumulation. **A** to **C)** RP–HPLC analysis of HMW GS **A)**, LMW GS **B)** and gliadin **C)** contents from mature WT and *gcn5* mutant seeds. Data are the mean ± SD of *n* = 3 replicates. Different lowercase letters indicate statistically significant differences (*P* < 0.05) between different genotypes as determined by one-way ANOVA. **D)** The positions of the primer sets (arrows) used in the ChIP assay relative to the transcriptional start sites (TSSs) are shown (*top*). ChIP–qPCR (*bottom*) assay of relative GCN5 enrichment at the *Glu-1Bx*, *Glu-1By*, *Glu-1Dx*, and *Glu-1Dy* promoters and gene bodies in WT versus *gcn5* mutants. Data are the mean ± SD of *n* = 3 replicates. Statistically significant differences between means are indicated by **, *P* < 0.01, as determined by Student's *t* test. **E, F)** ChIP–qPCR assay of relative H3K9ac enrichment **E)** and H3K14ac enrichment **F)** at the *Glu-1Bx*, *Glu-1By*, *Glu-1Dx*, and *Glu-1Dy* promoters in WT versus *gcn5* mutants. Data are the mean ± SD of *n* = 3 replicates. Statistically significant differences between means are indicated by **, *P* < 0.01, as determined by Student's *t* test. **G)** Genome browser views of H3K9ac marks at *Glu-1Bx*, *Glu-1By*, *Glu-1Dx*, and *Glu-1Dy* in WT, *gcn5-aaBBdd* and *gcn5-Aabbdd*. **H)** EMSA with recombinant CAMTA2 DNA-binding domain (amino acids 1 to 200) fused to MBP with biotin-labeled probes, unlabeled competitor probes and mutant competitor probes (MT-competitor) from the promoters of *Glu-1Bx* (top) and *Glu-1Dx* (bottom). The sequences of the probes and mutant competitors are shown. “+”, “−” and triangles indicate the presence, absence and increasing concentrations of the indicated probes, proteins or competitors. **I)** The positions of the primer sets (arrows) used in the ChIP assay relative to the transcriptional start sites (TSSs) are shown (*top*). ChIP–qPCR (bottom) assay of relative CAMTA2 enrichment at the *Glu-1Bx*, *Glu-1By*, *Glu-1Dx*, and *Glu-1Dy* promoters and gene body in WT and the *camta2*–*4* mutant. Data are the mean ± SD of *n* = 3 replicates. Statistically significant differences between means are indicated by **, *P* < 0.01, as determined by Student's *t* test. **J)** ChIP–qPCR assay of relative GCN5 enrichment at the *Glu-1Bx*, *Glu-1By*, *Glu-1Dx*, and *Glu-1Dy* promoter in WT and the *camta2*–*4* mutant. Data are the mean ± SD of *n* = 3 replicates.

GCN5 positively regulate the expression of *Glu* genes encoding HMW glutenin (Guo et al. 2015). Here, we profiled HMW glutenin contents in mature WT and *gcn5* mutant seeds using reversed-phase high-performance liquid chromatography (RP–HPLC). All quantified HMW glutenin subunits (GS) including Glu-1Bx, Glu-1By, Glu-1Dx, and Glu-1Dy decreased in *gcn5* mutants compared to WT (Fig. 7A), and no statistically significant differences in low-molecular-weight (LMW) GS were detected (Fig. 7B). By contrast, gliadin levels were slightly increased in *gcn5* mutants (Fig. 7C). *gcn5* mutants accumulated less dry gluten and had slightly lower SDS sedimentation volumes (Supplementary Fig. S11, A and B), an important indicator of processing and end-product quality indicating inferior end-use quality with the loss of GCN5. ChIP–qPCR showed GCN5 enrichment in the promoters of *Glu-1Bx*, *Glu-1By*, *Glu-1Dx*, and *Glu-1Dy* was attenuated in *gcn5* mutants (Fig. 7D), suggesting that GCN5 directly regulates these *Glu* genes. Moreover, H3K9ac and H3K14ac levels at *Glu-1Bx*, *Glu-1By*, *Glu-1Dx*, and *Glu-1Dy* were reduced in *gcn5* mutants determined by ChIP–qPCR (Fig. 7, E and F) and CUT&Tag assay (Fig. 7G), respectively. The transcripts of these 4 *Glu* genes were also decreased in *gcn5* mutants compared with WT in 20 DAP endosperm (Supplementary Fig. S11C). These data indicate that GCN5 regulates *Glu* expression by mediating the H3K9ac and H3K14ac levels in their respective promoter regions.

Considering the physical interaction between CAMTA2 and GCN5, we searched for a CAMTA2 binding motif in the promoter regions (2.5 kb upstream of the translational start site) of *Glu* genes. The binding of CAMTA2–MBP to the promoters of *Glu-1Bx* and *Glu-1Dx* was confirmed by EMSA (Fig. 7H). ChIP–qPCR showed that enrichment of CAMTA2 in the *Glu-1Bx*, *Glu-1By*, *Glu-1Dx*, and *Glu-1Dy* promoters was strongly attenuated in *camta2-4*, as expected (Fig. 7I). We assayed the expression of genes encoding SSPs in *camta2* knockout lines and saw 13 were downregulated compared to WT (Supplementary Fig. S12A), suggesting that CAMTA2 could directly bind to the promoters of SSP genes and positively regulated their expression. Moreover, we found that SPA, a transcription factor that positively regulates the expression of SSP genes (Ravel et al. 2014), was downregulated in *camta2* mutants compared to WT (Supplementary Fig. S12B), suggesting that CAMTA2 potentially modulates SSP genes by regulating SPA. To verify whether GCN5 is recruited to select *Glu* promoters by the transcription factor CAMTA2, ChIP–qPCR using an anti-GCN5 antibody in WT and *camta2-4* endosperm at 20 DAP was performed. GCN5 enrichment in the promoters of *Glu-1Bx*, *Glu-1By*, *Glu-1Dx*, and *Glu-1Dy* was unchanged (Fig. 7J), suggesting different regulatory mechanisms operate for CAMTA2 and GCN5 in starch versus SSPs accumulation. Unlike *gcn5* mutants, the HMW GS 1Bx, 1By, 1Dx. and 1Dy showed statistically nonsignificant changes in 2 *camta2* knockout lines (Supplementary Fig. S11D). In addition, there were statistically nonsignificant differences in LMW GSs and gliadin levels between the *camta2* knockouts and WT (Supplementary Fig. S11, D and E), suggesting that CAMTA2 might affect SSP genes at the level of transcription, but not the abundance of their respective protein products.

## Discussion

GCN5 is a well-known histone acetyltransferase responsible for lysine acetylation of histones in promoters of target genes through interactions with transcription factors involved in plant development (Gan et al. 2021). Several major transcription factors are now known to cooperatively activate gene expression by recruiting GCN5. For example, GCN5 interacts with PLATZ5 to confer powdery mildew resistance in wheat through activating a convergence point between pathogen- and effector-triggered immunity (Song et al. 2022). GCN5 regulates the transcription of *G1* and *PSBR1* through interacting with NACL to enhance thermotolerance in wheat (Lin et al. 2022). Here, we report that GCN5 and CAMTA2 positively and cooperatively regulate wheat grain size and weight. We found that knockdown of *GCN5* leads to smaller kernels (Fig. 1, A, C, and E) and less starch (Fig. 1F) and RNA-seq indicated that 21 genes related to starch biosynthesis were downregulated in *gcn5*. One possibility is that GCN5 directly regulates the expression of genes related to starch synthesis. For example, *Sus2* and *SBEIc* are directly bound by GCN5 to regulate acetylation levels in their promoters. The second possibility is that GCN5 indirectly affects the gene expression. For example, NF-YB1, a transcription factor interacting with MADS29 to regulate starch content during grain development in wheat (Liu et al. 2023), was downregulated in *gcn5* mutants, indicating that GCN5 may regulate the expression of starch biosynthesis genes by regulating NF-YB1.

Previous studies have demonstrated that GCN5 is accountable for the acetylation of H3K9 and H3K14 in different species such as Arabidopsis, rice, wheat, maize, yeast, and mammals. In this study, we saw that the overall H3K9ac levels were decreased in the *gcn5* mutants, while the H3K14ac total levels remained unchanged through immunoblot analysis. In both *gcn5-aaBBdd* and *gcn5-Aabbdd* mutants, only *GCN5* was downregulated and other HAT genes exhibited no change in expression levels compared to the WT (Supplementary Fig. S1C). These results indicated that only *GCN5* was affected in *gcn5* mutants. We also used an anti-H3K14ac antibody for ChIP–qPCR and saw a decrease in the level of H3K14ac at the promoter region of starch biosynthesis genes *Sus2* and *SBEIc*. It is possible that H3K14 level in some loci or genes were changed in *gcn5* mutants without affecting the global H3K14ac levels.

**Figure 8.**
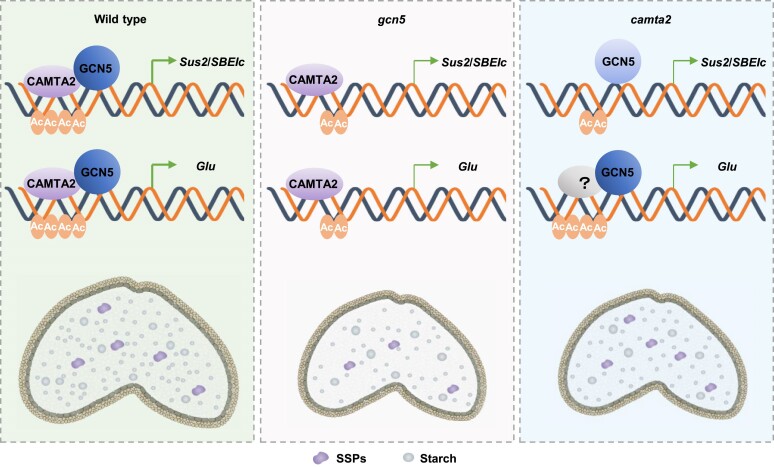
Model for GCN5 and CAMTA2 in regulating starch accumulation and *Glu* genes in wheat seeds. In WT seed, CAMTA2 binds to the CGCG motif in the promoters of *Sus2*, *SBEIc*, and *Glu* genes, and GCN5 interacts with CAMTA2 and establish H3K9ac and H3K14ac via recruitment by CAMTA2 to the promoters of *Sus2* and *SBEIc* genes and activates their expression. Loss of GCN5 reduces the expression of *Sus2*, *SBEIc*, and *Glu* by decreasing the levels of H3K9ac and H3K14ac, leading to smaller kernels, less starch, and less gluten. Mutation in CAMTA2 reduces the expression of *Sus2* and *SBEIc* by decreasing the GCN5 enrichment; *Glu* genes also downregulated in *camta2*, leading to smaller kernels, and less starch, while SSP abundance is unchanged. “?” indicates the potential involvement of additional transcription factors in regulating *Glu* genes expression. The thickness of arrows is proportional to gene expression levels, with greater thickness signifying higher expression.

Our IP–MS screen identified an interaction between GCN5 and CAMTA2. Field-grown *camta2* knockouts have similar phenotypes to field-grown *gcn5* mutants, including narrower grains (Fig. 4A and C), smaller thousand-grain weight (Fig. 4E), and less starch (Fig. 4F), indicating possible cooperative function between GCN5 and CAMTA2. A reduction of GCN5 enrichment and H3K9ac and H3K14ac levels in the promoters of *Sus2* and *SBEIc* was found in *camta2* null mutants, suggesting that GCN5 is recruited to the *Sus2* and *SBEIc* promoters to establish local histone acetylation on H3K9 and H3K14. Overall, we propose that CAMTA2 plays a dual role in activating expression of *Sus2* and *SBEIc* genes by directly binding to their promoters and recruiting GCN5 to modulate histone acetylation during seed development (Fig. 8). Starch biosynthesis in wheat is controlled by several transcription factors that regulate genes related to starch metabolism, such as bZIP28, RSR1, and NAC019 (Kang et al. 2013; Song et al. 2020; Huang et al. 2021; Gao et al. 2021, 2023). *PBF/LYS3* was also downregulated in the *camta2* mutant, suggesting that CAMTA2 might modulate starch biosynthesis genes by regulating the expression of *PBF/LYS3*. Indeed, transcription factors do not usually act alone, instead functioning in complexes in eukaryotes (He et al. 2023). Whether GCN5–CAMTA2 interacts with bZIP28 and RSR1 to regulate starch biosynthesis is an avenue we are pursuing as part of a wider interest in starch biosynthesis.

Wheat HMW genes significantly affect the dough processing quality and might be regulated by various epigenetic modification and transcription factors (Xiao et al. 2022; Zhang et al. 2023). GCN5 might target HMW glutenin genes by interacting with GAMyb to establish H3K9 and H3K14 acetylation in their promoters (Guo et al. 2015). In our study, we provide evidence that all HMW GS including Glu-1Bx, Glu-1By, Glu-1Dx, and Glu-1Dy were reduced in *gcn5* mutants, accompanied by lower gluten contents and SDS sedimentation volume, with downregulation of their respective transcripts. ChIP–qPCR revealed these *Glu* genes are targets of GCN5, with reduced H3K9ac and H3K14ac marks in their promoters in *gcn5* mutants. These data indicate that GCN5 regulates *Glu* genes by mediating the H3K9ac and H3K14ac levels in their promoters. CAMTA2 can directly bind to the CGCG motif present in the promoters of *Glu* genes to activate their expression. Thirteen SSP genes are downregulated in *camta2* knockouts. Furthermore, the SSP activator SPA (Ravel et al. 2014), was downregulated in *camta2* mutants and contains CGCG motifs in its promoter region, suggesting that CAMTA2 might also regulate SSP genes through *SPA*. Unlike in *gcn5* mutants, we found no statistically significant changes in the levels of quantified HMW GS, LMW GS, and gliadin levels. Fine-tuned regulation of gene expression involves multiple levels. In *camta2* mutant, the expression of the SSP genes was downregulated, yet the protein levels remain unchanged, which may be attributed to translational regulation. Adjustments in translation efficiency, such as optimization of the initiation and elongation processes, could maintain stable protein synthesis despite transcriptional downregulation (Verma et al. 2019). In addition, enhanced protein stability, achieved through post-translational modifications like phosphorylation, also contributes to maintain a constant protein abundance amid transcriptional fluctuations (Hu et al. 2023). Moreover, GCN5 enrichment in promoters of *Glu* genes was unchanged in *camta2* knockout lines. Therefore, we propose a model to describe the differences in how GCN5 and CAMTA2 regulate genes related to starch biosynthesis and *Glu* members (Fig. 8). In WT seeds, CAMTA2 directly binds to the CGCG motif in the promoters of *Sus2*, *SBEIc*, and *Glu* genes. GCN5 interacts with CAMTA2 to establish H3K9ac and H3K14ac via recruitment of CAMTA2 to the promoters of *Sus2*, *SBEIc*, and *Glu* genes and activates their expression to ensure the normal accumulation of starch and SSPs. Conversely, in *gcn5* mutants, the inactivation of GCN5 results in the downregulation of *Sus2*, *SBEIc*, and *Glu* genes by decreasing the levels of H3K9ac and H3K14ac, leading to smaller kernels, less starch, and less gluten. Mutation of CAMTA2 reduces the expression of *Sus2*, *SBEIc*, and *Glu* genes, leading to smaller kernels and less starch, while SSP abundance is unchanged in *camta2* knockout lines. We have obtained only *gcn5* and *camta2* single mutants, and the evidence for their cooperative regulation of storage proteins accumulation is insufficient. In the future, we will obtain double mutants and conduct phenotypic analyses to further substantiate this conclusion. Genetic complementation will validate the mechanism by which GCN5 and CAMTA2 cooperatively regulate the accumulation of storage proteins.

Since they were first characterized as transcription activators in 2000, CAMTAs are conserved in plants (Yang and Poovaiah 2000). Fifteen genes encoding CAMTAs have been identified in wheat (Yang et al. 2020), but until now, the functional characterization of these genes is limited and their roles in wheat seed development remain hitherto unknown. Our findings further the understanding of CAMTA function in wheat. CAMTA2 is involved in starch accumulation and influences grain size. Of the known calcium receptors, calmodulins have received the most attention and regulate a cellular response by interacting with calmodulin-binding proteins, including transcription factors—with CAMTAs among them. GW5, a calmodulin-binding protein in rice, acts as a positive regulator of BR signaling to regulate grain width and weight (Liu et al. 2017). Although additional functional evidence supporting the contribution of *CAMTA2* to grain yield is needed*, CAMTA2* represents a candidate for regulating wheat grain size and weight and can be exploited in molecular-breeding programs. In addition, *CAMTA2-A^H3^* with stronger binding to regulatory elements in the *Sus2* promoter positively correlates with grain size and weight. Importantly, 33.1% of sequenced modern cultivars carry *CAMTA2-A^H3^*, whereas this allele was detected in only 20.6% of the landraces examined, suggesting that this allele has been selected during modern breeding but has not yet been widely exploited in elite cultivars and represents a marker to use in classical breeding programs relying on natural variation.

## Materials and methods

### Plant materials and growth conditions

All wheat (*T. aestivum*) plants used for the phenotypic analysis were cultivated in an experimental field at China Agricultural University in Beijing (40°08′15″ N, 116°11′24″ E) during the growing seasons of 2022 and 2023. WT c.v. Fielder, *gcn5*, and *camta2* seeds were planted in a block containing rows 1.5 m long at a spacing of 20 cm in the field. Three identical blocks were planted. For gene expression analysis, IP and Co-IP assay, chromatin IP, and quantitative real-time PCR, whole seeds of WT, *gcn5*, and *camta2* at 4 DAP and endosperm at 10 DAP, 15 DAP, 20 DAP, 25 DAP, and 30 DAP were harvested from plants grown in the field. The endosperm was obtained by manual dissection of grains. For each sample, seeds and endosperm were collected from 3 different plants and were mixed to represent 1 biological replicate and at least 3 replicates were used. The samples were immediately frozen in liquid nitrogen and stored at −80 °C. And wheat plants used to propagate seeds were cultivated in a greenhouse at a relative humidity of 40% and 26/20 °C day/night temperatures with a 16-h light/8-h dark cycle. For wheat protoplast isolation, wheat plants were grown in the glasshouse at 26 °C in darkness, and 2-wk-old etiolated wheat seedlings were used. *N. benthamiana* plants, used for Co-IP and BiFC assays, were grown in a glasshouse at 22 °C under a 16-h/8-h light/dark cycle for 4 to 6 wk before infiltration.

### Production of *camta2* knockout mutants

A sgRNA was designed based on the first exon sequence of *CAMTA2* using the E-CRISP Design website (http://www.e-crisp.org/E-CRISP/designcrispr.html) and inserted into the expression cassette of the pBUE411 vector (Xing et al. 2014). The construct vector was transformed in wheat cultivar Fielder via *Agrobacterium*-mediated (EHA105) transformation following the method previously described (Medvecká and Harwood 2015).

### Phenotypic analyses

Thousand-grain weight, grain length, and width were determined using a camera-assisted phenotyping system (Hangzhou Wanshen Detection Technology Co., Ltd.). Eight replicates were used, comprising over 300 grains per replicate. The total starch contents of WT, *gcn5*, and *camta2* seeds were determined using Megazyme Total Starch Assay Kit (Megazyme; catalog no.: KTSTA-50A). SDS sedimentation values and dry gluten content were determined as previously described (Chen et al. 2019). The sample (∼100 mg) is added to the 2 mL test tube, and RP–HPLC analysis was performed as previously described (Gao et al. 2021). The starch WT and *gcn5* mutant lines were extracted as previously described (Chen et al. 2022), and the amounts of A-type (∼20 *µ*m) starch granules and B-type (∼5 *µ*m) starch granules were analyzed by laser particle size analyzer (Mastersizer). The mature seeds of WT and *gcn5* mutant lines were cut out, and the images of starch granules were taken with a Hitachi S-3400N scanning electron microscope (Tokyo, Japan). Three biological repeats were measured.

### RNA-seq and data analysis

Three biological replicates were performed for each sample. Total RNA was extracted from the endosperm of WT, *gcn5-aaBBdd*, and *gcn5-Aabbdd* at 25 DAP and *camta2-2* and *camta2-4* at 25 DAP using TransZol Plant (TransGen Biotech, ET121-01). RNA-seq libraries were a TruS prepared with eq RNA Sample Preparation Kit v2 (Illumina) and sequenced on NovaSeq 6000 platform. (IWGSC RefSeq v1.1) using STAR (Dobin et al. 2013). DESeq2 v1.24.0 was used for differential expression gene analysis (Love et al. 2014) and genes with log2(fold change) ≥ 0.75 and false discovery rate (FDR) < 0.05 were identified as differential expression genes. Significantly enriched GO categories of up- and downregulated genes [adjusted *P*-value (FDR) < 0.05] were identified using ClusterProfiler (Yu et al. 2012).

### RT–qPCR

Total RNA was extracted from seeds at 4 DAP and endosperm at 10 DAP, 15 DAP, 20 DAP, 25 DAP, and 30 DAP using TransZol Plant (TransGen Biotech, ET121-01). First-strand cDNAs were synthesized from 1 *μ*g of RNA using HiScript II Q RT SuperMix (Vazyme Biotech, R223-01) according to the manufacturer's instructions. For RT–qPCR assay, Taq Pro Universal SYBR qPCR Master Mix (Vazyme Biotech, Q712-02/03) was used. The reaction mixture was composed of 5 *µ*L SYBR Master Mix, 1 *µ*L cDNA template, and 0.2 mm primers in a final volume of 10 *µ*L. Amplification was performed using a CFX96 real-time system (BioRad), and *ACTIN* (TraesCS5B02G124100) was the reference gene. Differences in relative transcript levels were calculated using the C_t_ (2^−ΔCT^) method (Schmittgen and Livak 2008). RT–qPCR was performed as technical triplicates per sample. Three biological replicates were performed, with similar results; the results from 1 replicate are shown in the figures. Significant differences are indicated by *P < 0.05 and **P < 0.01, as determined by Student's *t* test. Primers are listed in Supplementary Data Set 8.

### ChIP–qPCR

ChIP was performed as previously described (Yang et al. 2016) with some modifications. For ChIP assays, ∼1.5 g of 20 DAP WT, *gcn5*, and *camta2* mutant endosperm was ground into powder in liquid nitrogen and cross-linked with 1% (*v*/*v*) formaldehyde in a vacuum desiccator for 10 min at 25 °C. The cross-linking reaction was stopped by the addition of 2 M glycine, followed by nucleus isolation and sonication with a Bioruptor Plus System (Qsonica). The anti-H3K9ac (Abcam, ab10812), anti-H3K14ac (Millipore, 07-353), anti-IgG (Abcam, ab190475), anti-GCN5 antibodies generated by Abmart (Guo et al. 2015), and anti-CAMTA2 generated by Abclonal were used for IP. The precipitated DNA was recovered using a Hieff NGS DNA Selection Beads (Yeasen, 12601ES56) and analyzed by qPCR. De-cross-linked DNA from the chromatin fraction before incubation with the antibody was used as the control (input sample). ChIP values were normalized to the input sample, and the enrichment was calculated based on the relative enrichment of IP with anti-GCN5, CAMTA2, H3K9ac, and H3K14ac antibodies compared to that with anti-IgG IP (Li et al. 2015). The means and standard deviations were calculated from 3 biological replicates and 3 technical replicates. Statistical significance was determined with Student's *t* test at *P < 0.05 and **P < 0.01. Primers are listed in Supplementary Data Set 8.

### CUT&Tag and data analysis

CUT&Tag was performed as previously described (Zhao et al. 2023). CUT&Tag assay was performed using endosperm isolated from 8 DAP WT, *gcn5-aaBBdd*, and *gcn5-Aabbdd* mutants. The experiment was performed with 2 biological replicates. Data analysis was performed as previously described (Zhang et al. 2024).

### IP assays

Total proteins were extracted from endosperm of WT, *gcn5*, and *camta2* knockout lines at 20 DAP as previously described (Gao et al. 2021). Nuclear proteins were extracted from endosperm of WT, *gcn5-Aabbdd ,*and *gcn5-aaBBdd* at 20 DAP using a nuclear and cytoplasmic protein extraction kit (Beyotime, P0027) according to the manufacturer's instructions, and immunoblot analysis was performed as previously described (Gao et al. 2021). Three biological replicates were performed. Antibodies anti-H3 (Abcam, ab1791), anti-H3K9ac (Abcam, ab10812), anti-H3K14ac (Millipore, 07-353), anti-H3K27ac (Millipore, 07-360), anti-H4ac (Millipore, 06-598), and H4K5ac (Millipore, 07-327), H4K12ac (Millipore, 07-595) were all used at 1:5,000 for immunoblot analysis. The anti-GCN5 antibodies generated by Abmart (Guo et al. 2015) and anti-CAMTA2 generated by Abclonal were used at 1:2,000 for immunoblot analysis.

### Co-IP assay in wheat endosperm

The endosperms of WT and *gcn5-Aabbdd* were ground into powder in liquid nitrogen and extracted using lysis buffer (50 mm Tris-HCl pH 7.5, 1 mm EDTA pH 8.0, 150 mm NaCl, 10 mm MgCl_2_, 10% *v*/*v* glycerol, 0.5% *v*/*v* NP-40, 5 mm DTT, and 0.1 mm PMSF with protease inhibitor cocktail, Roche, Switzerland). The solution was placed on ice for 10 min and centrifuged at 13,000 rpm at 4 °C for 10 min. Extracts were incubated at 4 °C overnight with anti-GCN5 antibody, and then protein A/G magnetic beads (Millipore, 3783542) were added and incubated for 4 h. Samples were washed 10 times with 1 mL lysis buffer. Eluted proteins were analyzed by 10% (*w*/*v*) SDS–PAGE and stained using a Protein Silver Stain Kit (Mei5 Biotechnology, MF329-01) according to the manufacturer's instructions. Gel bands were excised for IP–MS analysis by Beijing Genomics Institute using *gcn5-Aabbdd* as a control. After digestion, 10 *μ*L solution containing peptides was used for mass spectrometer detecting. The peptides were separated by the UltiMate3000 RSLCnano ultrahigh-performance liquid system and then analyzed by the Thermo Scientific Q Exactive mass spectrometer. The resulting MS data were processed using MASCOT2.3.0. Proteins identified by IP–MS as interacting with GCN5 are listed in Supplementary Data Set 4.

### Co-IP assay in *N. benthamiana* leaves

The *GCN5* ORF was amplified and cloned into the *XbaI* site of the pCAMBIA1300-GFP vector (Liu et al. 2021), while the *CAMTA2* ORF was cloned into the *SmaI* site of the pCAMBIA1300-MYC vector to produce CAMTA2–MYC vectors. Agrobacteria transformed with the fusion constructs were co-infiltrated into *N. benthamiana* leaves. Total proteins of *N. benthamiana* leaves were extracted using lysis buffer (50 mm Tris-HCl pH 7.5, 1 mm EDTA pH 8.0, 150 mm NaCl, 10 mm MgCl_2_, 10% *v*/*v* glycerol, 0.5% *v*/*v* NP-40, 5 mm DTT, 0.1 mm PMSF, 20 *µ*M MG132 with protease inhibitor cocktail, Roche, Switzerland). Lysates were incubated at 4 °C for 6 h with anti-GFP magnetic beads (Easybio, China) and washed 10 times with 1 mL lysis buffer. Eluted proteins were analyzed by 10% (*w*/*v*) SDS–PAGE, and immunoblot analysis was detected by immunoblotting with anti-GFP (diluted 1:2,000; TransGen Biotech, HT801-01) or anti-MYC (diluted 1:2,000; TransGen Biotech, HT101-01) antibodies. Three biological replicates were performed. The primers for Co-IP assay are listed in Supplementary Data Set 8.

### Y2H assay

The full-length *GCN5* open reading frame (ORF) was cloned into the *EcoRI* site of the pGBKT7 vector (Takara, Japan), while the full-length *CAMTA2* was cloned into the *EcoRI* site of the pGADT7 vector (Takara, Japan). Different combinations of the plasmids were transformed into yeast strain AH109, and the cells were grown on minimal medium (-Leu -Trp) at 30 °C for 2 d according to the manufacturer's instructions (Clontech). Cells were serially diluted and plated on minimal medium (-Leu -Trp -His -Ade) to test for possible interactions. Combinations of the T-antigen and P53 were used as a positive control. Three biological replicates were performed. The primers for Y2H used are listed in Supplementary Data Set 8.

### In vitro pull-down assay

The *GCN5* ORF was amplified and cloned into the *EcoRI/XbaI* sites of the pMAL-c2x vector (NEB, E8000S) in-frame with the *MBP* sequence. To generate His-tagged CAMTA2 protein, the *CAMTA2* ORF was amplified and cloned into the *EcoRI/SalI* sites of the pET-32a vector (Novagen). Recombinant proteins were expressed in *E.coli* Rosetta (DE3) and induced with 0.5 mm isopropyl-β-D-thiogalactopyranoside (IPTG) in lysogeny broth (LB) overnight at 16 °C. Recombinant protein was isolated following the manufacturer's instructions. For the MBP pull-down assay, different combinations of the *E. coli* lysates were incubated with Tris buffer (25 mm Tris-HCl pH 7.5, 50 mm NaCl, 1 mm DTT) for 5 h at 4 °C using high-affinity amylose resin (NEB, E8021L). The beads were washed 8 times with 1 mL Tris buffer + 1% *v*/*v* Triton X-100 and then eluted in 100 *µ*L 2× loading buffer [100 mm Tris-HCl pH 6.8, 20% (*v*/*v*) glycerol, 4% (*w*/*v*) SDS, and 10% (*v*/*v*) β-mercaptoethanol] at 100 °C for 10 min. Bound proteins were analyzed by 10% (*w*/*v*) SDS–PAGE, and immunoblot analysis as performed by immunoblotting with anti-MBP (diluted 1:5,000; TransGen Biotech, HT701-01) or anti-His (diluted 1:5,000; TransGen Biotech, HT501-01) antibodies. Three biological replicates were performed. The primers for in vitro pull-down assay are listed in Supplementary Data Set 8.

### BiFC assay

The full-length *GCN5* ORF and the truncated coding sequences were all cloned into the *BamHI/XbaI* sites of the N-YFP vector, while the full-length *CAMTA2* and the truncated coding sequences were cloned into the *BamHI/XbaI* sites of the C-YFP vector. Agrobacteria GV3101 containing different fusion constructs were co-infiltrated into *N. benthamiana* leaves. YFP signals were imaged 48 h after infiltration with a confocal microscope (LSM880; Carl Zeiss, Heidenheim, Germany; laser, 488 nm; intensity, 3.98%; collection bandwidth, 484 to 543 nm; gains, 600 to 800 v). Three biological replicates were performed. The primers for BiFC assay are listed in Supplementary Data Set 8.

### RNA in situ hybridization

For RNA in situ hybridization, developing seeds at 10 and 20 DAP were used and performed as previously described (Zhang et al. 2024). Three biological replicates were performed. The sense and antisense RNA probes are listed in Supplementary Data Set 8.

### Subcellular localization

The *CAMTA2* ORF was amplified and cloned into the *XbaI* site of the pCAMBIA1300-GFP vector (Liu et al. 2021). *A. tumefaciens* GV3101 containing different fusion constructs were co-infiltrated into *N. benthamiana* leaves. The fused protein OsSPL16–RFP [SQUAMOSA PROMOTER BINDING (SBP) domain-containing transcription factor] was used as a positive control for nuclear localization (Gao et al. 2021). GFP and RFP signals were imaged 48 h after infiltration. GFP signals were imaged with a confocal microscope (LSM880; Carl Zeiss, Heidenheim, Germany; laser, 488 nm; intensity, 2%; collection bandwidth, 475 to 574 nm; gains, 600 to 800 v). RFP signals were imaged with a confocal microscope (LSM880; Carl Zeiss, Heidenheim, Germany; laser, 543 nm; intensity, 2%; collection bandwidth, 578 to 640 nm; gains: 500 to 800 v). Three biological replicates were performed. The primers for Subcellular localization are listed in Supplementary Data Set 8.

### EMSA

The coding sequences of *CAMTA2-A-BD^H1^* (amino acids 1 to 200) and *CAMTA2-A-BD^H3^* were cloned into the pMAL-c2x vector (NEB, E8000S) via *EcoRI/XbaI* sites to produce a MBP fusion protein. Recombinant protein was expressed in *E. coli* Rosetta (DE3) and induced with 0.5 mm IPTG in LB overnight at 16 °C and then purified using high-affinity amylose resin (NEB, E8021L). Oligonucleotide probes were synthesized and 5′ end-labeled with biotin (Invitrogen). Double-stranded probes were obtained by adding complementary oligonucleotides, boiling at 100 °C for 5 min and cooling to room temperature. EMSAs were carried out using a Light Shift Chemiluminescent EMSA Kit (no. 20148; Thermo Fisher Scientific) following the manufacturer's instructions. Proteins and probes were incubated in a 20 *µ*L solution supplemented with 1× binding buffer (100 mm Tris-HCl pH 7.5, 500 mm KCl, 10 mm DTT), 2.5% *v*/*v* glycerol, 5 mm MgCl2, 5 mm KCl, 50 ng/mL poly(dI-dC), 0.2 mm EDTA, and 0.05% *v*/*v* NP-40 at 25 °C for 20 min. The reaction mixtures were separated in a 6% native polyacrylamide gel and transferred to a nylon membrane. Three replications were performed for each assay.

### Transcriptional activation assay in wheat leaf protoplasts

Isolation and transfection of wheat leaf protoplasts were performed as previously described (Wen et al. 2023). To generate the effector construct GAL4-BD–CAMTA2, the coding sequence of *GAL4–DB* and *CAMTA2* were cloned into the *KpnI* site of pCAMBIA130 vector to produce GAL4–BD and GAL4–BD–CAMTA2. The pGreenII 0800-LUC contains an internal control gene from *Renilla reniformis* (REN), driven by a promoter with 5× the *GAL4* UAS sequence and a TATA box introduced as a reporter construct for GAL4 binding. The reporter and effector constructs were co-transformed into wheat leaf protoplasts and GAL4–BD as a control. Statistical significance was determined with Student's *t* test at *P* < 0.05. Three replicates were performed. The sequences of the primers are listed in Supplementary Data Set 8.

### Dual-luciferase transcriptional activity assay

To generate the reporter constructs, fragments corresponding to 2,500 bp upstream of the TSS of *Sus2* and *SBEIc* were amplified and cloned into *HindIII/KpnI* site of the pGreenII 0800-LUC vector (Hellens et al. 2005). The *CAMTA2* coding sequences were cloned into the *XbaI/SalI* site of the pCAMBIA1300-FLAG vector to produce the CAMTA2–FLAG vectors. CAMTA2–FLAG was used as the effector. The reporter and effector constructs were co-transformed into wheat leaf protoplasts. The transfected protoplasts were cultured in dark for 14 h at 22 °C. Firefly luciferase activity derived from the *Sus2_pro_:LUC* and *SBEIc_pro_:LUC* reporters and REN under the control of the *35S* promoter (*35S_pro_:REN*) were quantified using the Dual-Luciferase Reporter Assay system (Promega) according to the manufacturer's instructions. Normalized data are presented as the ratio of luminescent signal intensity for the *LUC* reporter versus internal control reporter (*35S_pro_*:*REN*) from 3 independent biological samples. Statistical significance was determined with Student's *t* test at *P* < 0.01. The sequences of the primers are listed in Supplementary Data Set 8.

### Statistical analysis

Three independent biological replicates were performed in each experiment. Student's *t*- est was applied for comparisons between two sample groups. For multiple comparisons, a significance analysis was determined by one-way ANOVA using IBM SPSS Statistics 27. Error bars represent the SD of each set of data. Statistically significant differences were determined by one-way ANOVA or the Student's *t* test *P < 0.05 and **P < 0.01. Detailed statistical analysis data are shown in Supplementary Data Set 9.

### Accession numbers

Sequence data from this article can be found in the EMBL library (http://plants.ensembl.org/index.html) under the following accession numbers: *GCN5-A* (TraesCS1A02G138200), *GCN5-B* (TraesCSU02G003200), *GCN5-D* (TraesCS1D02G134200), *CAMTA2-A* (TraesCS4A02G407100), *CAMTA2-B* (TraesCS4B02G306300), and *CAMTA2-D* (TraesCS4D02G304500). RNA-seq and CUT&Tag data are available at the National Center for Biotechnology Information Sequence Read Archive (http://www.ncbi.nlm.nih.gov/sra) under accession number PRJNA1104432.

## Supplementary Material

koae261_Supplementary_Data

## Data Availability

The data underlying this article are available in the article and in its online supplementary material.

## Supplementary data

The following materials are available in the online version of this article.

**Supplementary Figure S1.** Immunoblot of GCN5 in *gcn5* lines.

**Supplementary Figure S2.** Phenotype of mature *gcn5* mutants.

**Supplementary Figure S3.** GO analysis of genes upregulated in 25 DAP endosperm in *gcn5* mutants.

**Supplementary Figure S4.** Heat map of starch metabolism genes downregulated in *gcn5* mutants.

**Supplementary Figure S5.** GCN5 physically interacts with CAMTA2.

**Supplementary Figure S6.** Organ and developmental time course of CAMTA2 expression.

**Supplementary Figure S7.** Generation of *camta2* mutants by gene editing.

**Supplementary Figure S8.** Heat map of starch metabolism genes downregulated in *camta2* mutants.

**Supplementary Figure S9.** EMSA of the recombinant CAMTA2–MBP (1 to 200 aa) and the predicted CG-1 DNA-binding domain (22 to 134 aa).

**Supplementary Figure S10.** KASP markers designed to separate the three *CAMTA2-A* haplotypes.

**Supplementary Figure S11.** Seed phenotypes of *gcn5* and *camta2* mutants.

**Supplementary Figure S12.** Heat map of SSP genes downregulated in *camta2* mutants.

**Supplementary Data Set 1.** List of differentially expressed transcripts in *gcn5* and the WT.

**Supplementary Data Set 2.** Expression of genes related to starch biosynthesis in *gcn5* and the WT.

**Supplementary Data Set 3.** List of genes with decreased H3K9ac levels in *gcn5* compared to WT.

**Supplementary Data Set 4.** Proteins identified by IP–MS may interact with GCN5.

**Supplementary Data Set 5.** List of differentially expressed transcripts in *camta2-4* and the WT.

**Supplementary Data Set 6.** Expression of genes related to starch biosynthesis in gcn5, camta2, and the WT.

**Supplementary Data Set 7.** The 445 wheat accessions used for haplotype analysis.

**Supplementary Data Set 8.** Primers used for gene expression analysis, vector construction, and ChIP–qPCR.

**Supplementary Data Set 9.** Detailed results of statistical analyses.

## Dive Curated Terms

The following phenotypic, genotypic, and functional terms are of significance to the work described in this paper:

FAD3 Gramene: AT2G29980
FAD3 Araport: AT2G29980

CBP60g Gramene: AT5G26920
CBP60g Araport: AT5G26920

grain width Planteome: TO:0000402

ABRE Gramene: Abscisic acid response element
ABRE Araport: Abscisic acid response element
