# Supplementary material for: The transcription factor CAMTA2 interacts with the histone acetyltransferase GCN5 and regulates grain weight in wheat
Source: Plant Cell. 2024 Sep 25;36(12):4895–913. doi: 10.1093/plcell/koae261 (PMC11638106; doi:10.1093/plcell/koae261)
Supplement: koae261_Supplementary_Data [file koae261_supplementary_data.zip › Supplementary Data.pdf]

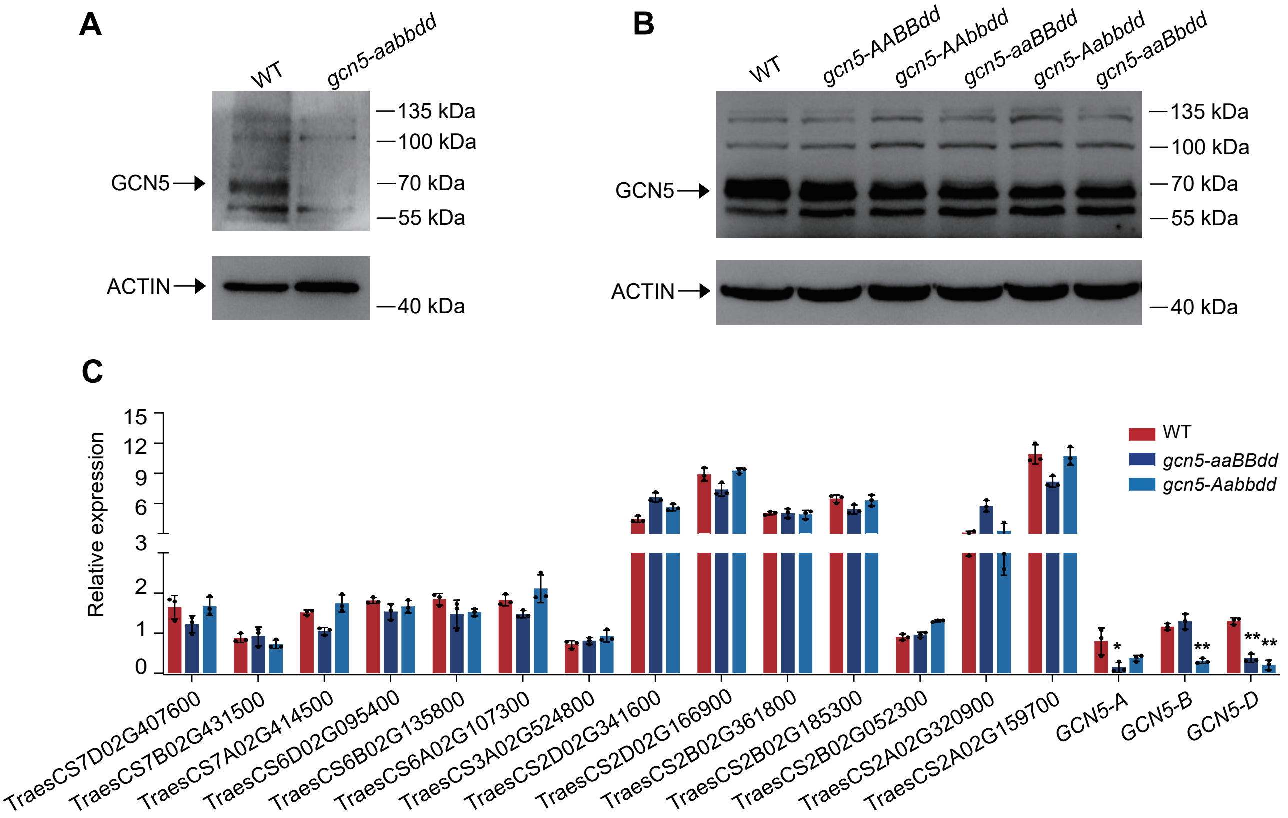

Supplementary Figure S1. Immunoblot of GCN5 in *gcn5* lines.

(A) Total soluble protein was extracted from leaves from the Wild type (WT) and *gcn5-aabbdd* and probed with anti-GCN5 antibody (top) and the anti-ACTIN antibody (bottom). Total proteins were loaded onto 10% (w/v) SDS–PAGE gels and ACTIN was used as a loading control. (B) Total soluble protein was extracted from endosperm tissue at 20 DAP from the indicated genotypes and probed with anti-GCN5 antibody (top) and the anti-ACTIN antibody (bottom) as a loading control. A representative result from three independent replicates is shown. Total proteins were loaded onto 10% (w/v) SDS–PAGE gels and ACTIN was used as a loading control. (C) FPKM of 17 HAT genes in WT, *gcn5-aaBBdd* and *gcn5-Aabbdd* endosperm at 25 DAP. Data are the mean ± standard deviation (SD) of n = 3 replicates. Statistically significant differences between means of genotypes were determined by Student's *t* test against the WT control and are indicated by *, $P < 0.05$; **, $P < 0.01$.

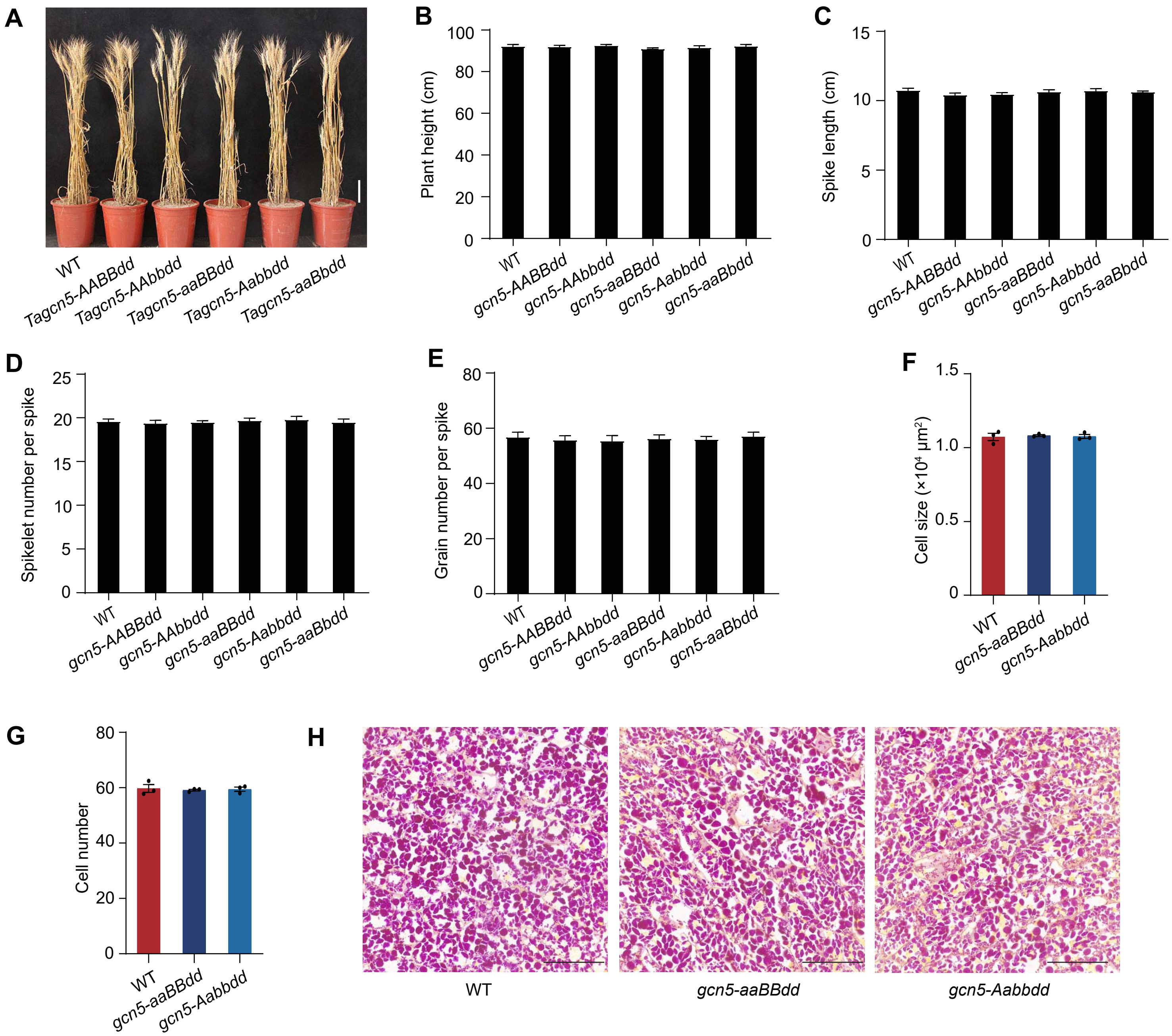

Supplementary Figure S2. Phenotype of mature *gcn5* mutants.

(A) Mature plants of WT and *gcn5* mutants under field conditions. Scale bar = 10 cm. (B–E) Plant height, spike length, spikelet number per spike and grain number per spike in WT and *gcn5* mutants. Data are the mean ± SD of n = 10 replicates. The effect of genotype on statistically significant differences in means for y-axis traits was determined by one-way ANOVA (*P* < 0.05). (F, G) Cell size and cell number in WT and *gcn5* mutants. Data are the mean ± SD of n = 3 replicates. (H) Histological analysis of developing endosperm at 15 DAP in WT and *gcn5* mutants. Scale bar = 200 μm.

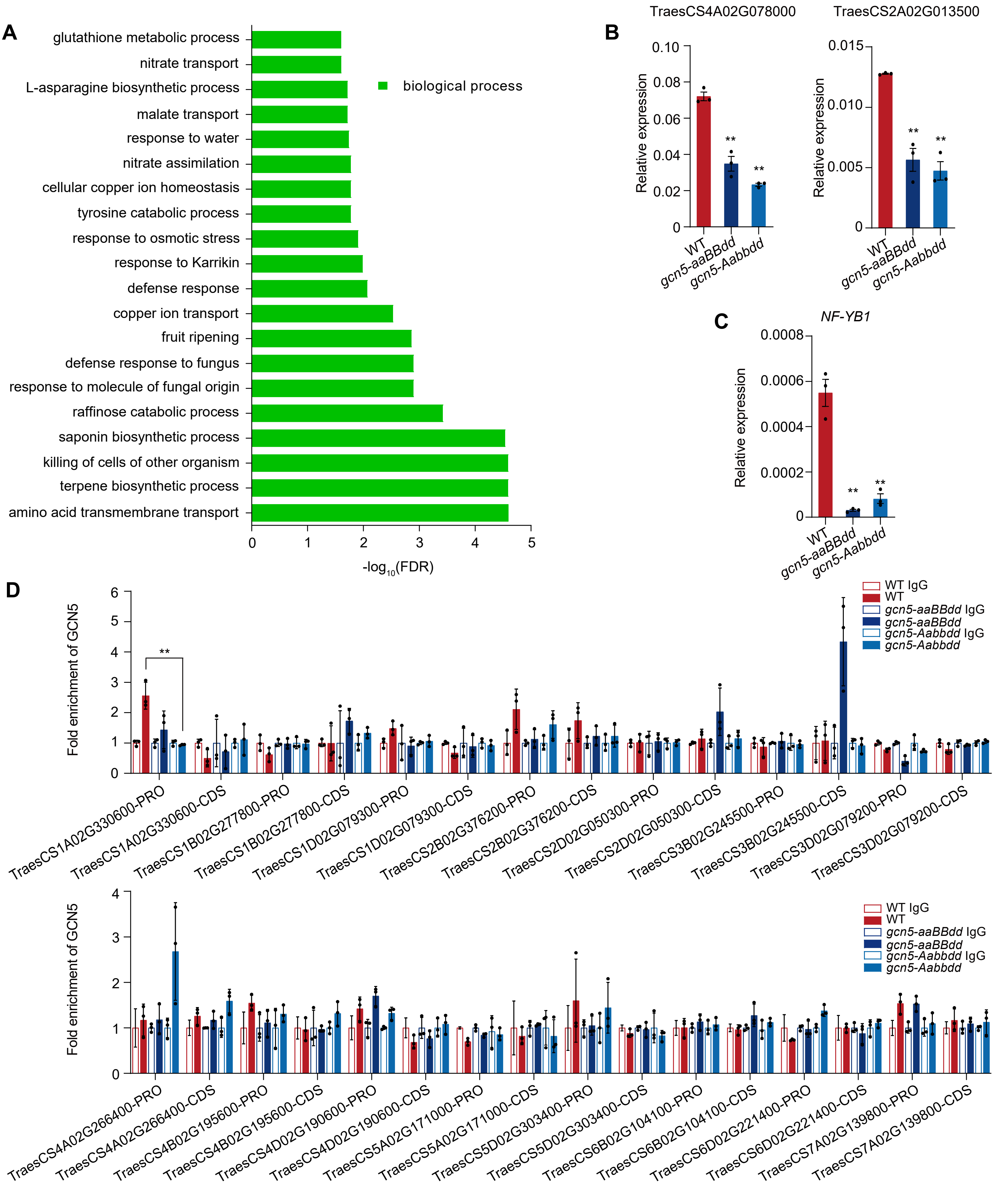

Supplementary Figure S3. GO analysis of genes up-regulated in 25 DAP endosperm in *gcn5* mutants.

(A) GO analysis of genes up-regulated in 25 DAP endosperm in *gcn5* mutants. Only genes differentially expressed in both *gcn5-aaBBdd* and *gcn5-Aabbdd* mutants were considered. *P*-values were adjusted by the Benjamini–Hochberg correction and only statistically significant GO categories (False Discovery Rate [FDR] < 0.05) are shown. (B) RT–qPCR of two genes (TraesCS4A02G078000 and TraesCS2A02G013500) associated with BR biosynthesis in WT, *gcn5-aaBBdd* and *gcn5-Aabbdd* endosperm at 25 DAP. Data are the mean ± SD of n = 3 replicates. Statistically significant differences between means of genotypes were determined by Student's *t* test against the WT control and are indicated by **, *P* < 0.01. (C) RT–qPCR of *NF-YB1* in WT, *gcn5-aaBBdd* and *gcn5-Aabbdd* endosperm at 25 DAP. Relative expression was normalized to *ACTIN*. Data are the mean ± SD of n = 3 replicates. Statistically significant differences between means of genotypes were determined by Student's *t* test against the WT control and are indicated by **, *P* < 0.01. (D) ChIP–qPCR assay of relative GCN5 enrichment at the promoters and gene bodies of 15 genes evenly distributed over wheat chromosomes in WT versus *gcn5* mutants. Two pairs of primers were designed for each gene to target promoter and gene-body regions. The enrichment was calaulated based on the relative enrichment in anti-GCN5 compared with anti-IgG. Data are the mean ± SD of n = 3 replicates. Statistically significant differences between means of genotypes were determined by Student's *t* test against the WT control and are indicated by **, *P* < 0.01.

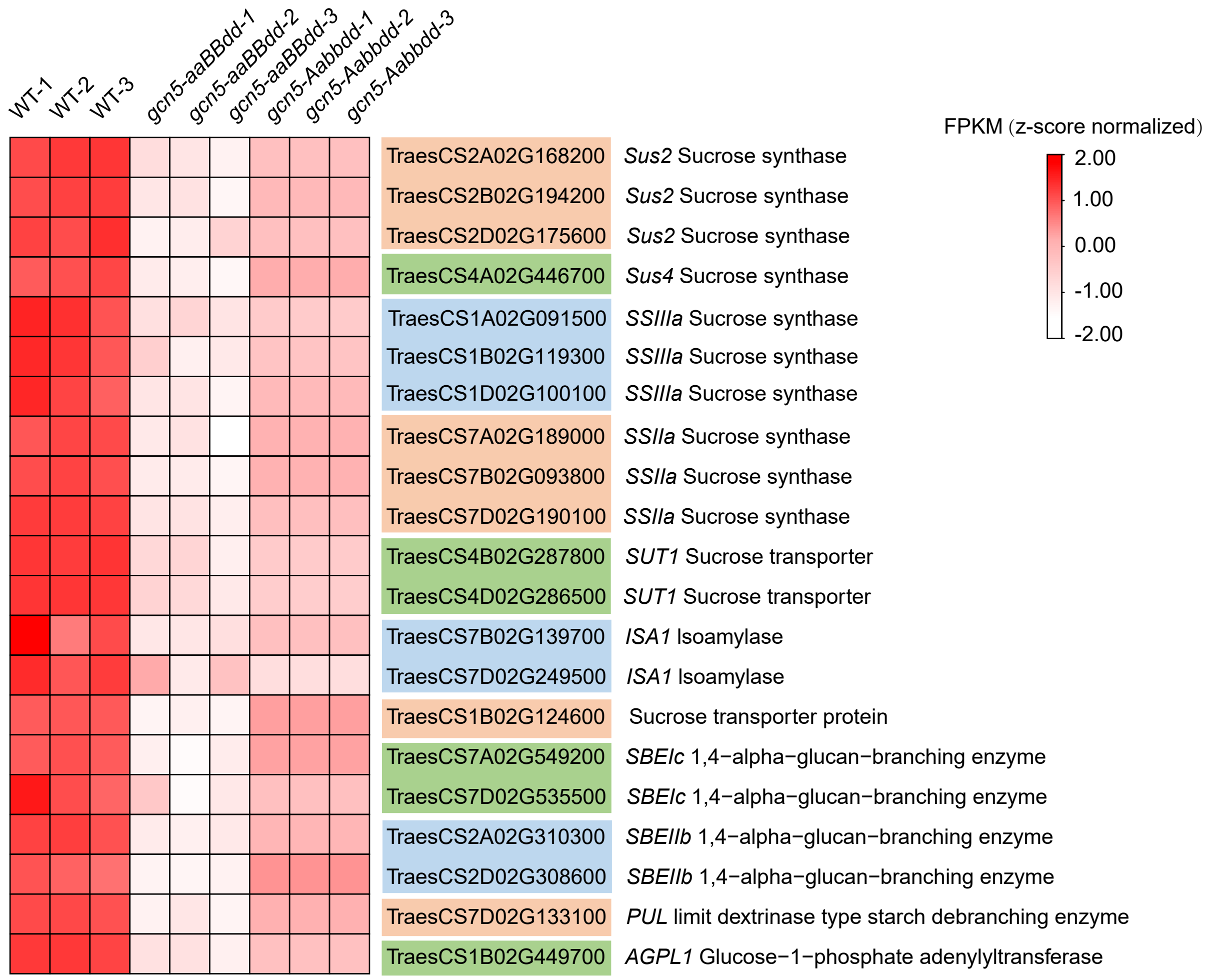

Supplementry Figure S4. Heat map of starch-metabolism genes down-regulated in *gcn5* mutants.

Heat map illustrating the FPKM-based expression patterns of starch-metabolism genes in 25 DAP endosperm. Only genes differentially expressed in both *gcn5-aaBBdd* and *gcn5-Aabbdd* mutants relative to WT were considered. Colored rectangles indicate the respective homoeologs for each gene. Heat color represent the expression level from white (weaker) to red (stronger). The heatmap was drawn using the R package ‘pheatmap’, and FPKM values of three biological replicates were normalized using the ‘scale = row’ parameter from the pheatmap package.

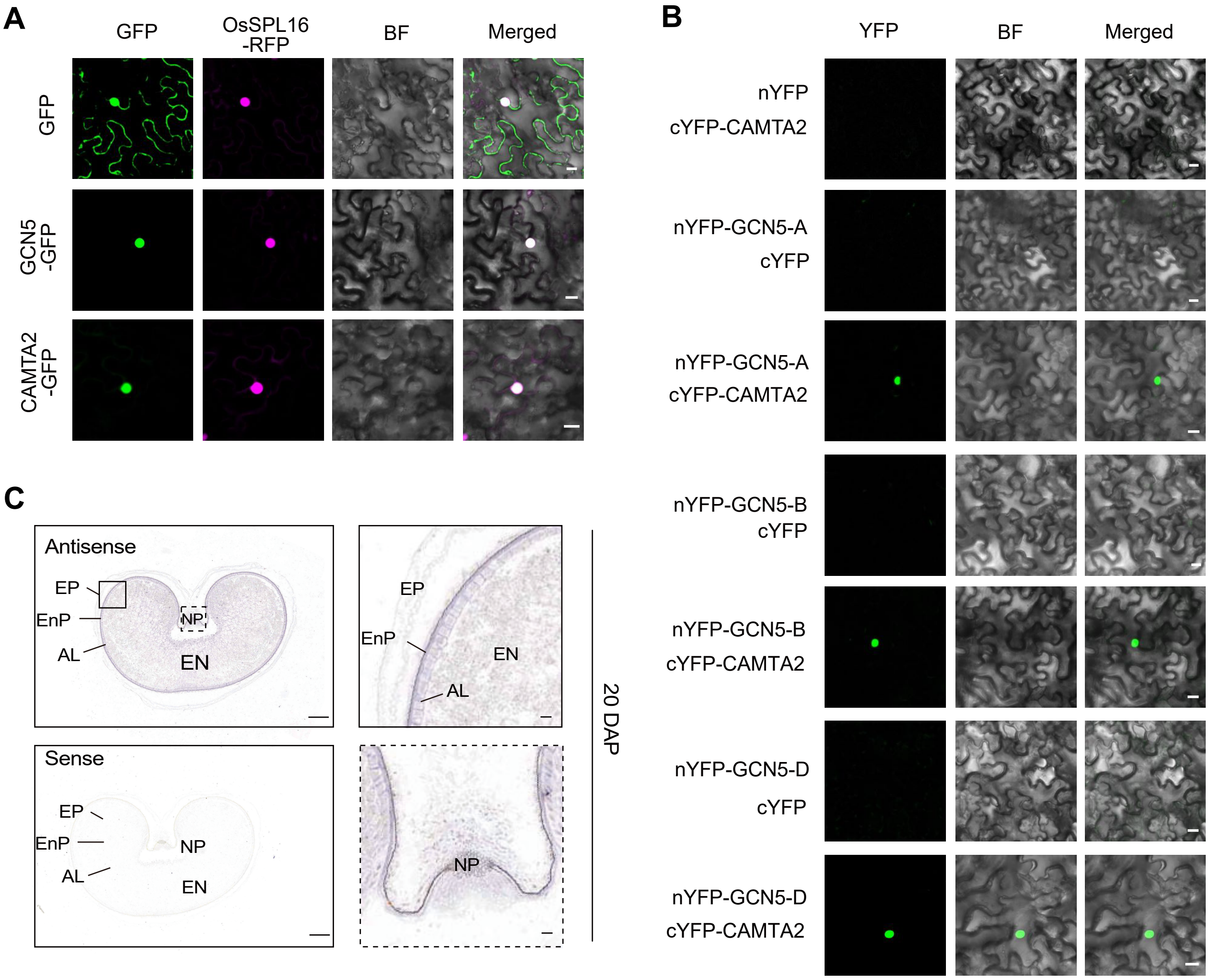

Supplementary Figure S5. GCN5 physically interacts with CAMTA2.

(A) Subcellular localization of GCN5 and CAMTA2 fused to GFP. Fusion proteins were transiently expressed in *N. benthamiana* leaves. SPL16–RFP (SQUAMOSA PROMOTER BINDING (SBP) domain-containing transcription factor) fused to RFP was used as a positive control for nuclear localization (Gao et al., 2021). Scale bars = 20 μm. (B) BiFC assay to verify the interactions between the three GCN5 homoeologs and CAMTA2. Scale bars = 20 μm. (C) Representative images of *in situ* hybridization assays with 20 DAP transverse seed sections hybridized with antisense and sense *CAMTA2* probes. EP: exocarp; EnP: endocarp; AL: aleurone layer; NP: nucellar projection; En: endosperm. Scale bars = 500 μm.

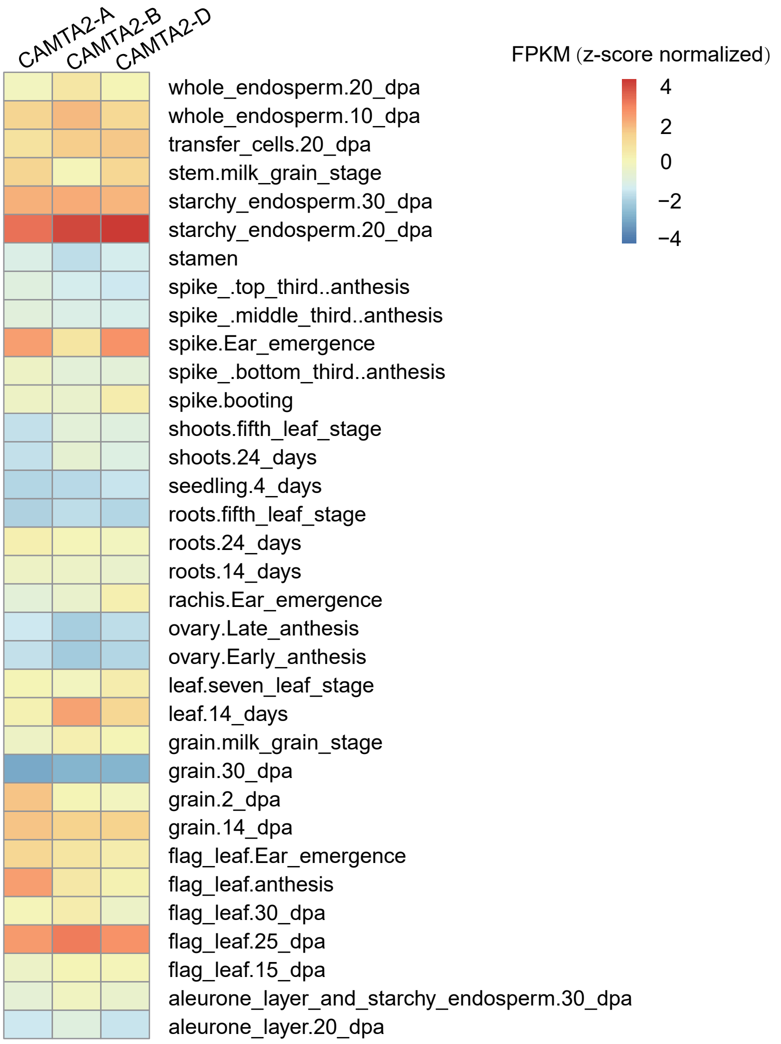

Supplementary Figure S6. Organ and developmental time course of *CAMTA2* expression.

The spatial and temporal expression patterns of *CAMTA2* homoeologs was assessed using publicly available RNA-seq data from a range of tissues, covering vegetative and reproductive stages. The heat map represents relative expression level from blue (weaker) to red (stronger).

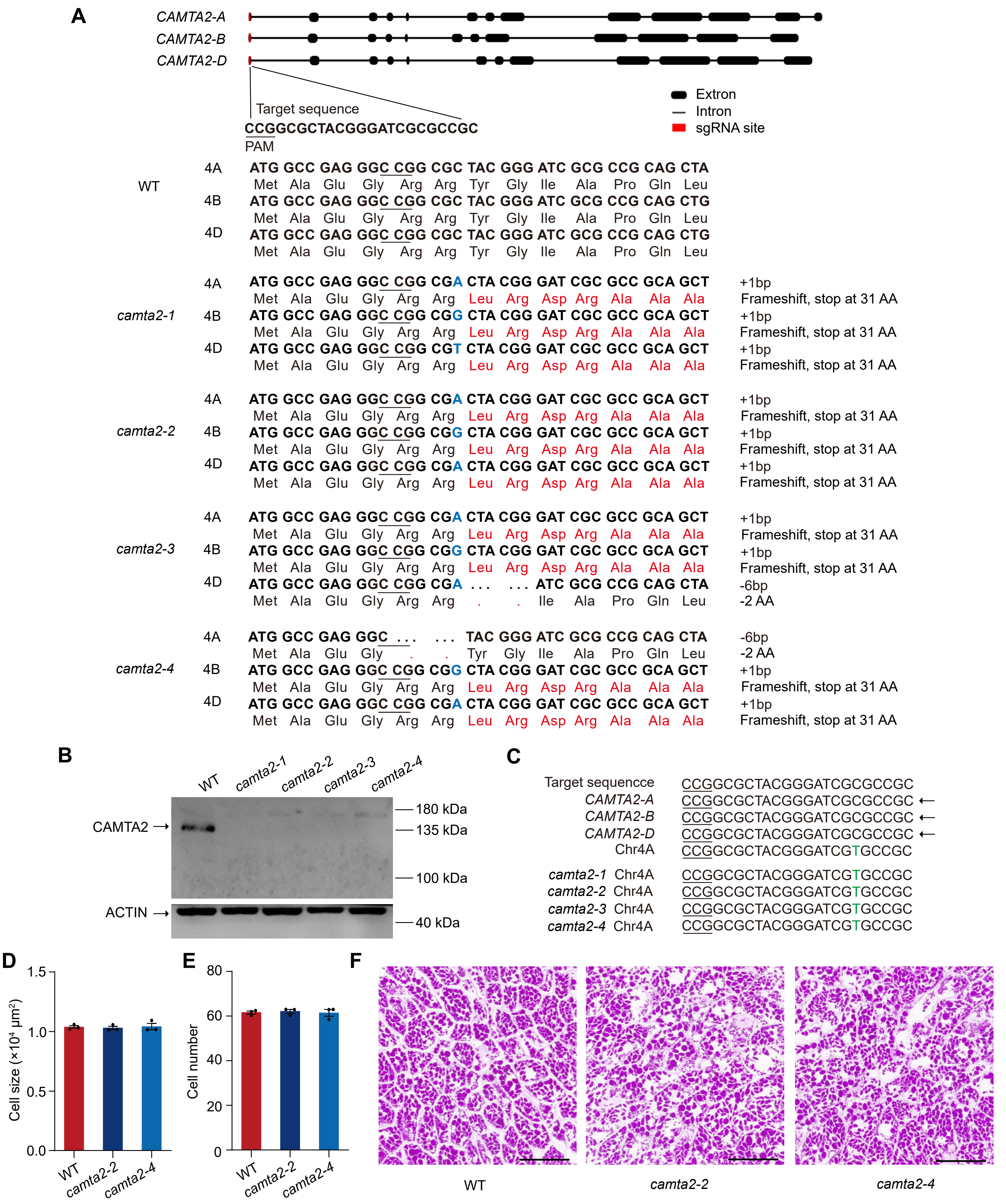

Supplementary Figure S7. Generation of *camta2* mutants by gene editing.

(A) Upper panel: Sequence of the sgRNA used to target a region in exon 1 encoding the DNA-binding domain conserved among the three *CAMTA2* homoeologs. The protospacer-adjacent motif (PAM) sequence is underlined. Bottom panel: Sequences of WT and the four recovered *camta2* mutants around the sgRNA binding site in the three *CAMTA2* homoeologs. Nucleotide insertions are indicated in blue. '.' indicate deletions. Numbers indicate the number of base pairs of the insertions and deletions. Amino-acid sequences of WT and the four recovered *camta2* mutants were shown under each DNA sequence. The amino-acid changes are highlighted in red. (B) Western blot against CAMTA2 (expected size = 119.32 kDa) in 20 DAP endosperm isolated from WT, *camta2–1*, *camta2–2*, *camta2–3 and camta2–4* mutants sequenced in panel A. ACTIN was used as a loading control. A representative result from three independent replications is shown. Total protein was loaded onto 10% (w/v) SDS–PAGE gels. (C) Sequence analysis of potential off-target events. The PAM sequence is underlined, different bases are shown in green. The black arrows indicate the sequences of *CAMTA2-A*, *CAMTA2-B* and *CAMTA2-D* of WT. No base mutations, insertions, or deletions are observed in the predicted off-target sites. (D, E) Cell size and cell number in WT and *camta2* mutants. Data are the mean ± SD of n = 3 replicates. (F) Histological analysis of developing endosperm at 15 DAP in WT and *camta2* mutants. Scale bar = 200 μm.

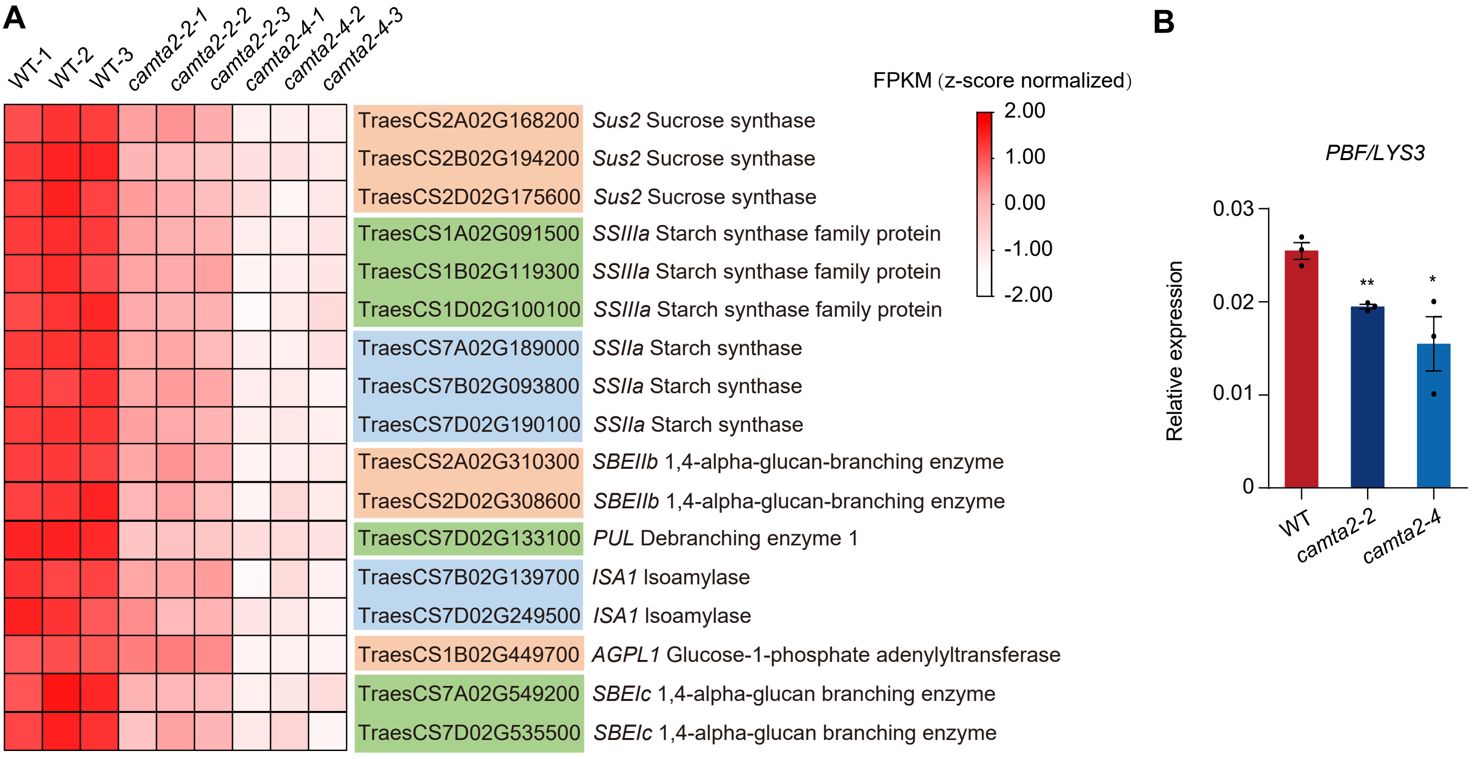

Supplementary Figure S8. Heat map of starch-metabolism genes down-regulated in *camta2* mutants.
(A) FPKM-based expression patterns of starch-metabolism genes in 25 DAP endosperm of WT, *camta2–2* and *camta2–4*. Colored rectangles demarcate the respective homoeologs for each gene. The heat map represents the relative expression level from white (weaker) to red (stronger). The heatmap was drawn using the R package 'pheatmap' and FPKM values of three biological replicates were normalized using the 'scale = row' parameter from pheatmap. (B) RT–qPCR of *PBF/LYS3* in WT, *camta2–2* and *camta2–4* endosperm at 25 DAP. Relative expression was normalized to *ACTIN*. Data are the mean ± SD of n = 3 replicates. Statistically significant differences are indicated by *, $P < 0.05$; **, $P < 0.01$, as determined by Student's *t* test.

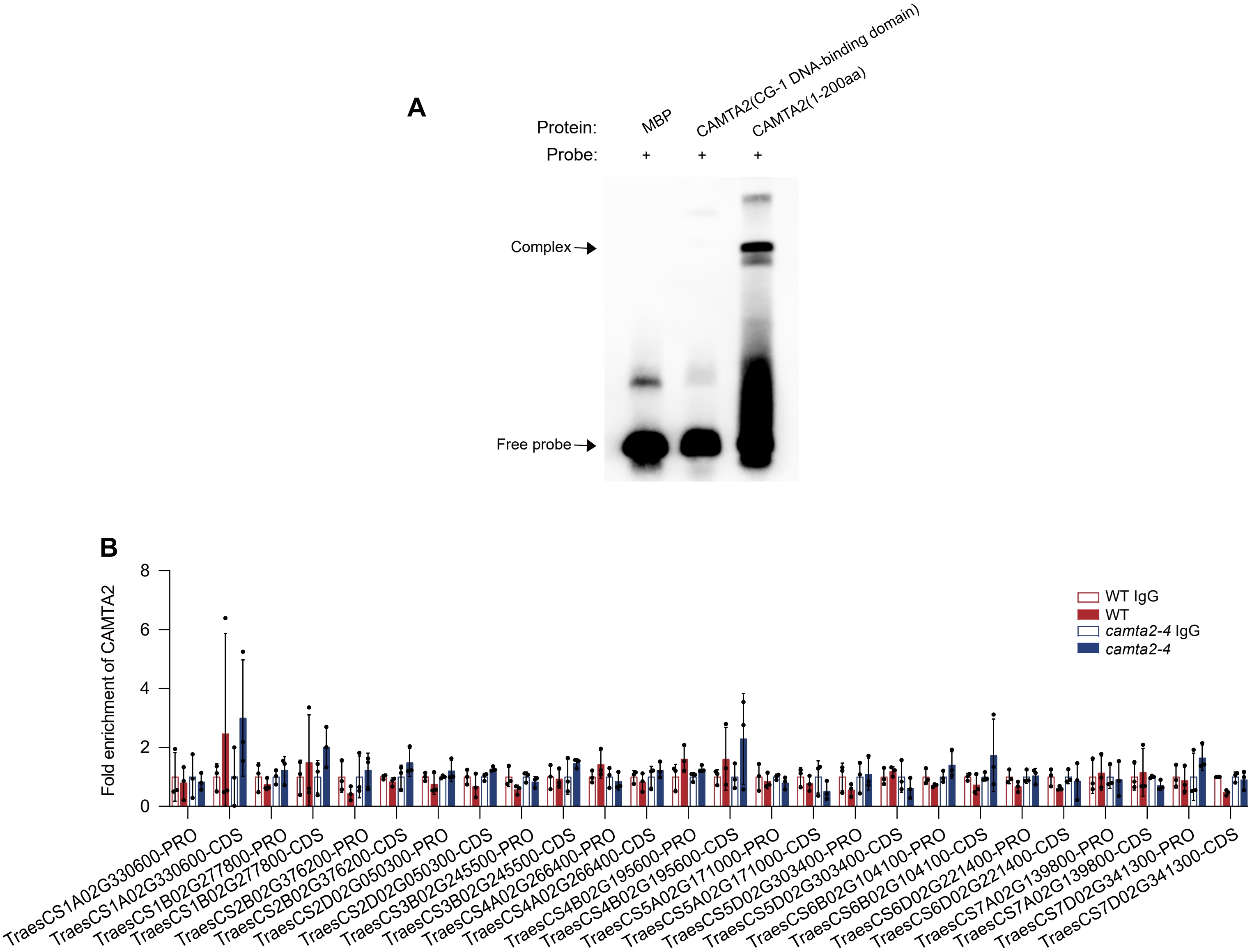

Supplementary Figure S9. EMSA of the recombinant CAMTA2–MBP (1–200 aa) and the predicted CG-1 DNA-binding domain (22–134 aa). (A) Biotin-labeled probes were derived from the promoter of *Sus2*. 4 μg protein of CAMTA2–MBP (22–134 aa) and CAMTA2–MBP (1–200 aa) were loaded onto gels for EMSA. (B) ChIP–qPCR assay of relative CAMTA2 enrichment at the promoters and gene bodies of 13 genes evenly distributed over wheat chromosomes in WT versus *camta2* mutants. Two pairs of primers were designed for each gene to target promoter and gene-body regions. The enrichment was calaulated based on the relative enrichment in anti-CAMTA2 compared with anti-IgG. Data are the mean ± SD of n = 3 replicates.

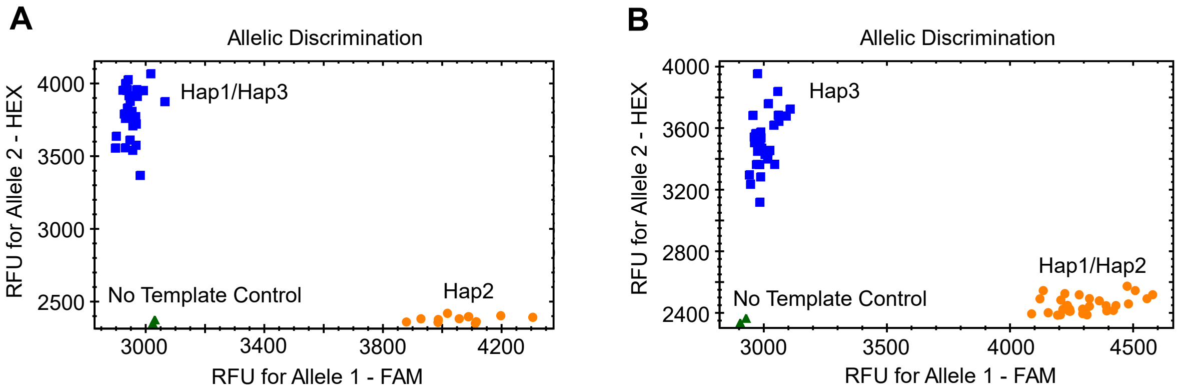

Supplementary Figure S10. Kompetitive allele-specific PCR (KASP) markers designed to separate the three *CAMTA2-A* haplotypes. (A) Kompetitive allele-specific PCR (KASP) marker based on the SNP of *CAMTA2-A* between Hap2 and the other two haplotypes at amino-acid position 116. Blue dots indicate Hap1/Hap3 and orange dots indicate Hap2. Green dots indicate the no-template control. (B) Kompetitive allele-specific PCR (KASP) marker based on the SNP of the *CAMTA2-A* between Hap3 and other two haplotypes at amino-acid position 194. Blue dots indicate Hap3 and orange dots indicate Hap1/Hap2 respectively. Green dots indicate the no-template control.

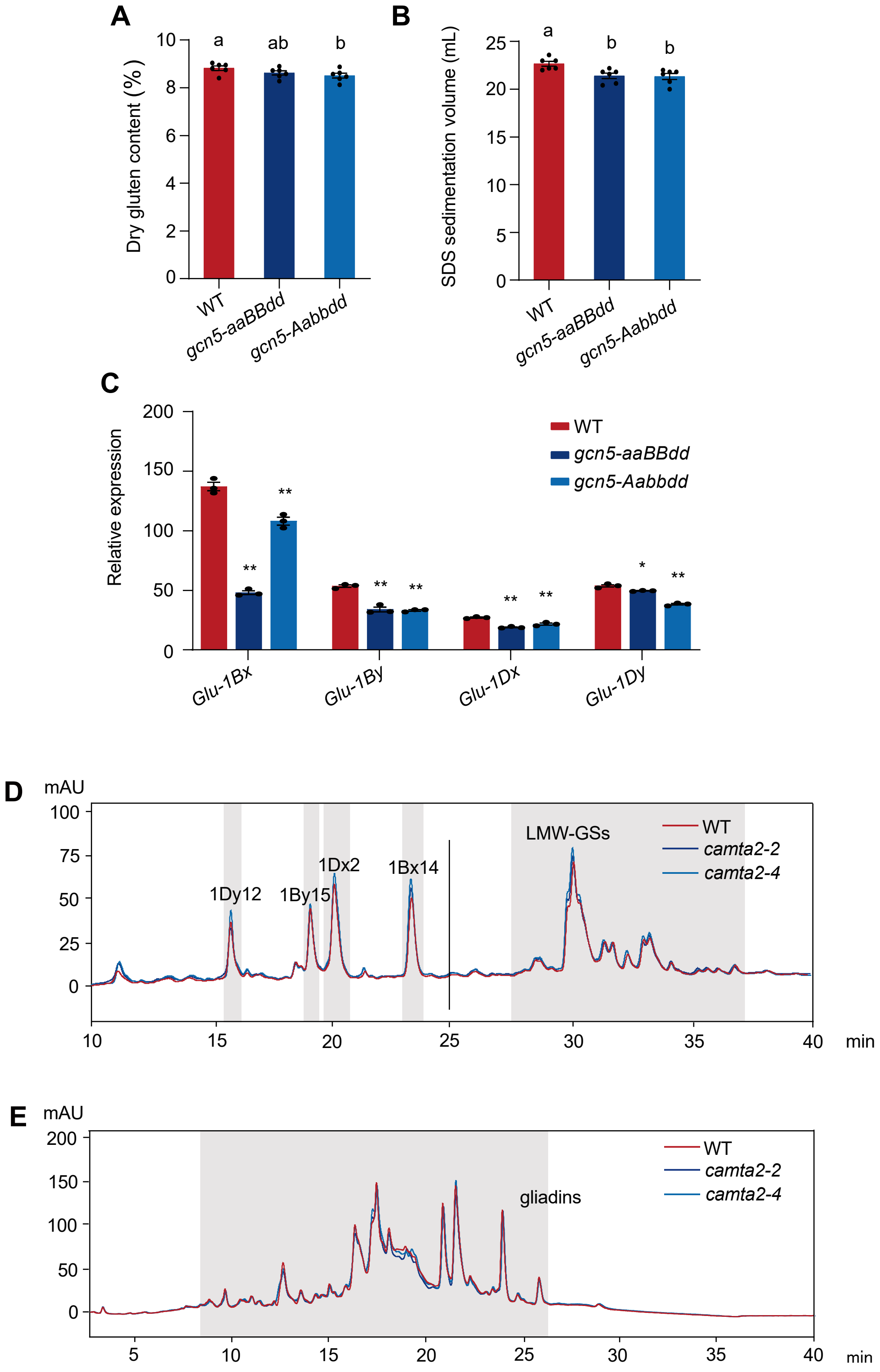

Supplementary Figure S11. Seed phenotypes of *gcn5* and *camta2* mutants.

(A) Dry-gluten contents and (B) SDS sedimentation volume in WT and *gcn5* mutants. Data are the mean ± SD of n = 6 replicates. Different lowercase letters indicate a statistically significant difference (P < 0.05) between different genotypes as determined by one-way ANOVA. (C) RT–qPCR of *Glu-1Bx*, *Glu-1By*, *Glu-1Dx and Glu-1Dy* expression in 20 DAP endosperm isolated from WT, *gcn5-aaBBdd* and *gcn5-Aabbdd*. Relative expression was normalized to *ACTIN*. Data are the mean ± SD of n = 3 replicates. Statistically significant differences between means of genotypes were determined by Student's *t* test against the WT control and are indicated by *, P < 0.05; **, P < 0.01. (D, E) Representative RP–HPLC chromatograms of HMW-GS and LMW-GS (D), and gliadin (E) in seed extracts from WT, *camta2-2* and *camta2-4*. The light gray shading indicates the chromatographic peaks corresponding to various seed extracts.

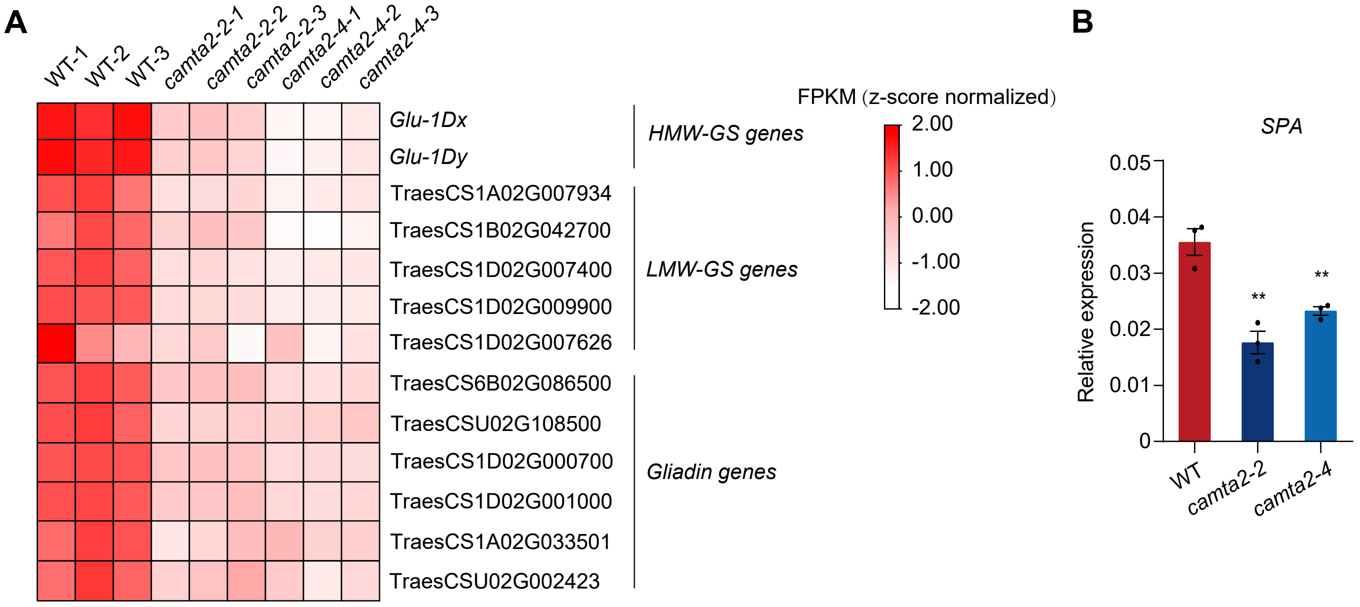

Supplementary Figure S12. Heat map of seed-storage-protein genes down-regulated in *camta2* mutants.

(A) FPKM-based expression patterns of seed-storage proteins in 25 DAP endosperm of WT, *camta2–2* and *camta2–4*. Heat maps represent the relative expression level from white (weaker) to red (stronger). The heatmap was drawn using the R package ‘pheatmap’ and FPKM values of three biological replicates were normalized using the ‘scale = row’ parameter from the pheatmap package. (B) RT–qPCR of *SPA* in WT, *camta2–2* and *camta2–4* endosperm at 25 DAP. Relative expression was normalized to *ACTIN*. Data are the mean ± SD of n = 3 replicates. Statistically significant differences are indicated by **, $P < 0.01$, as determined by Student’s *t* test.
