# Supplementary material for: The transcription factor CAMTA2 interacts with the histone acetyltransferase GCN5 and regulates grain weight in wheat
Source: Plant Cell. 2024 Sep 25;36(12):4895–913. doi: 10.1093/plcell/koae261 (PMC11638106; doi:10.1093/plcell/koae261)
Supplement: koae261_Supplementary_Data [file tpc.01206.2023-s15.pdf › tpc.01206.2023-s15.pdf]

THE PLANT CELL

Wheat grain weight is regulated by the histone acetyltransferase GCN5 interacting with the CAMTA2 transcription factor

Ruijie Zhang, Kexin An, Yujiao Gao, Zhaoheng Zhang, Xiaobang Zhang, Xue Zhang, Vincenzo Rossi, Yuan Cao, Jun Xiao, Mingming Xin, Jinkun Du, Zhaorong Hu, Jie Liu, Huiru Peng, Zhongfu Ni, Qixin Sun, and Yingyin Yao

Review Timeline:

Submission Date:	15-Dec-2023
Editorial Decision:	22-Jan-2024
Revision Received:	29-Apr-2024
Editorial Decision:	05-Jun-2024
Revision Received:	22-Jul-2024
Accepted:	09-Aug-2024

Prof. Yingyin Yao
China Agricultural University
Plant genetics and breeding
Beijing 100193
China

Dear Dr. Yao:

We have received reviews of your manuscript entitled "Wheat grain weight is regulated by the histone acetyltransferase GCN5 by physical interaction with the CAMTA2 transcription factor.." Thank you for submitting your work to The Plant Cell. The editorial board agrees that the work you describe is substantive, falls within the scope of the journal, and may become acceptable for publication, pending revision and potential re-review.

We ask you to pay particular attention to R1's critique and request for ChIP-seq and R2's comments on weak GCN5-CAMTA2 functional connection in preparing your revision.

We should stress that we are reluctant to see manuscripts undergoing multiple rounds of revision and would be unlikely to offer you more than one chance to satisfy the reviewers.

The editors noted that although this manuscript is a good fit for the scope of the journal, we have decided it is not a good fit for the focus issue on Translational research, so it will be considered as a standard Research Article.

Please contact us if there are ambiguous comments or if you wish to discuss the revision.

Given the nature of the comments, we are offering you 90 days from when we have issued this decision to complete the revision. If a revision is not returned within this time frame, and if you have not been granted an extension, we will withdraw the manuscript, which will leave you free to submit the work elsewhere. If you need an extension, we encourage you to contact us at any point before the 90 days have passed.

When you are ready to submit the revised version, please upload a highlighted copy that indicates all changes made in response to the editor and reviewer recommendations. Include an itemized list of all changes made in response to each of the reviewer's suggestions in the "Response to Reviewers" section; please note that reviewers do not have access to your cover letter, nor was this decision letter shared with them.

Thank you for the privilege of reviewing your work. We look forward to receiving your revised manuscript.

Sincerely,

The Plant Cell Editorial Board

Please note the following:

-The Plant Cell now requires authors to complete and submit an author revisions checklist upon submission of a revised manuscript. The aim of the checklist is to aid authors in preparing a high-quality manuscript, facilitate the review and assessment of revised manuscripts, and help to ensure that journal standards are maintained across the board. If your manuscript is accepted, the completed checklist will be published as supplemental material attached to the article online. Please download a copy of the checklist (pdf fillable form) at this link, for submission with your revised manuscript: https://tpc.msubmit.net/html/Author_Revisions_Checklist.pdf.

-Supplemental materials should be restricted to large datasets and tables, presentation of replicates, and validation of reagents, methods, or genotypes. Any data that are used to support the major claims must be in the main manuscript. Supplemental figure legends must indicate what figure in the main manuscript is supported by the supplemental data presented. Please justify how each of the supplemental figures meet the criteria.

-Sampling methods and nature of "biological replicates" should be described precisely (i.e. different plants, parts of plants, pooled tissue, independent pools of tissue, sampled at different times, etc), along with a clear description of and rationale for any statistical analyses conducted. The reader should know exactly what was sampled; what forms the basis of the calculation of any means and statistical parameters reported. This is also necessary to ensure that proper statistical analysis was conducted.

-Want to add this revision deadline to your calendar? Click below!

-- Reviewer comments:

Reviewer #1 (Comments for the Author):

In their manuscript, Zhang et al., studied the function of GCN5 and CAMTA2 in the regulation of wheat grain size and weight. Using classical genetics, molecular biology and proteomics approaches authors found that CAMTA2 interacts with GCN5. Authors proposed that this complex activates SUSy2A and SBEIc by directly binding to their promoters during wheat endosperm development. The overall results are interesting; however some conclusions are not supported by the data presented.
1. Authors claimed that their "results collectively indicate that GCN5 contributes to the regulation of SUSy2A and SBEIc expression by mediating H3K9ac levels in their promoters." This is a key result of the paper but to my view the ChIP-QPCR is not enough to prove that GCN5 bind directly those two genes. ChIP-QPCR is a biased technique that those days serves to confirm ChIP-seq which is completely unbiased. To my view since authors have an antibody that works in ChIP, they must perfom a ChIP-seq.
2. GCN5 in Arabidopsis, yeast and mammals (DOI 10.1074/jbc.M111.329417 ; doi.org/10.1371/journal.pone.0054896 ; DOI:10.1111/J.1365-313X.2007.03264.X ; doi: 10.1093/nar/gkaa369.) it is clearly demonstrated that the primary site of acetylation by Gcn5 is H3K14. However, in the results displayed by the authors it seems that in wheat it is not the case. (i) Is it really GCN5 that is mutated or another HAT? How authors can explain that in Arabidopsis, yeast and mammals GCN5 target H3K14 and not in wheat?
3. As for GCN5, a ChIP-seq for CAMTA2 is mandatory.
4. Authors showed in their model that GCN5 and CAMTA2 binds chromatin at the same position. To prove that authors need to perform a ChIPre ChIP experiment.

Reviewer #2 (Comments for the Author):

A major breeding goal is to boost grain weight in wheat. Of them, starch acts as a predominant role in determining grain weight. However, the improvement of starch content is a major challenge for modern agriculture due to the limited understanding of the molecular regulatory network by which starch is synthesized and accumulated in developing endosperm. In this manuscript, the authors generated a GCN5 mutant using the CRISPR/Cas9 strategy, and focused on the grain phenotypic defect of the gcn5 mutant. The further experiment revealed that the gcn5 mutation differentially affects the expression of genes associated with starch and storage protein biosynthesis through directing binding to their promoters. More strikingly, they also identified the transcription factor CAMTA2 as the interacting partner of GCN5 to cooperatively affect starch but not storage proteins. The topic of this manuscript is interesting for readers. Overall, the experiments in this manuscript are well conducted, and the data is well present. However, the cellular and genetic data are relatively weak. Below, please see my suggestions for revising or improving the manuscript:

Major comments:

1. To understand the cellular mechanism of GCN5 and CAMTA2 to regulate wheat grain size and weight, the cellular section analyses of wheat grain need to be done. Besides, from the RNA sequencing data, the affected cell expansion genes, cell division genes and hormone related genes need to be selected for RT-qPCR to analyze the molecular mechanism of GCN5 and CAMTA2 to regulate wheat grain size and weight.

2. The authors claimed that GCN5 and CAMTA2 work cooperatively to regulate storage substance accumulation. The only data to support this proposition is the phenotypic data of single mutants. The reviewer suggests the authors to generate a double mutant to enhance their conclusion. If possible, complementation test should also be supplemented for knock-out mutants mentioned in this manuscript.

3. The reviewer noticed that numerous genes associated with storage starch and proteins are also seriously down-regulated in the gcn5 or camta2 mutants. The possible explanations should be discussed.

4. Given that GCN5 has been widely reported in plants, the novelty of this manuscript should be further enhanced through revising the text to highlight the light spot.

Minor comments:
1. The meanings of the sentence "gcn5 knocks have down-regulated Glu transcripts levels encoding high molecular weight (HMW) glutenin and to lower levels of HMW glutenin protein, gluten contents and SDS sedimentation volume" is not well clear, please revise this sentence to depict clearly its meanings.

2. In Figure 2D, the ratio of different H3 acetylation to H3 proteins need be calculated to well display the different H3

acetylation change pattern.

3. From Supplemental Figure 1c, this claim "No visible phenotypic differences were detected in gcn5 mutant mature plants relative to wild type, including plant height, tiller number and spike number" is not well supported. The Authors need to collect the statistical data of plant height, tiller number and spike number to support their claim.

4. In Figure 3D,E, the font in the right boxes is too small to see, please use bigger fonts.

5. Seed-storage proteins genes were down-regulated in camta2 mutants, why the seed-storage proteins are unaffected in camta2 mutants? What is the possible mechanism?

6. English should be improved by a professional editor. In addition, there are some typos that should be checked throughout the manuscript.

Dear Editor and Reviewers,

We wish to thank you all for your time, assistance, and suggestions for the improvement of our manuscript. We have revised the manuscript '**Wheat grain weight is regulated by the histone acetyltransferase GCN5 by physical interaction with the CAMTA2 transcription factor**' (Manuscript ID TPC2023-RA-01206), wherein we have done our best to robustly address all the reviewers' concerns and suggestions.

Below we provide point-by-point responses (in dark brown) to the reviewers' comments. The indicated line numbers refer to the revised manuscript wherein revisions are highlighted by track changes.

Reviewer #1 (Comments for the Author):

In their manuscript, Zhang et al., studied the function of GCN5 and CAMTA2 in the regulation of wheat grain size and weight. Using classical genetics, molecular biology and proteomics approaches authors found that CAMTA2 interacts with GCN5. Authors proposed that this complex activates SUSy2A and SBEIc by directly binding to their promoters during wheat endosperm development. The overall results are interesting; however some conclusions are not supported by the data presented.

1. Authors claimed that their "results collectively indicate that GCN5 contributes to the regulation of SUSy2A and SBEIc expression by mediating H3K9ac levels in their promoters." This is a key result of the paper but to my view the ChIP-QPCR is not enough to prove that GCN5 bind directly those two genes. ChIP-QPCR is a biased technique that those days serves to confirm ChIP-seq which is completely unbiased. To my view since authors have an antibody that works in ChIP, they must perfom a ChIP-seq.

Response: We thank the reviewer for this suggestion; however, addressing this has proven difficult. We have conducted multiple rounds of ChIP-seq using anti-GCN5

and anti-CAMTA2 antibodies over the past three months. We also explored various conditions to establish the most effective protocol toward mitigating bias. This included the type of cross-linking reagents used, namely formaldehyde and DSG (disuccinimidyl glutarate), the duration of the cross-linking process with 10 and 15 min as the respective intervals, the sonication time, which was varied between 25 and 30 min to optimize chromatin shearing. However, the experiment did not produce satisfactory results, as shown in the figures below. Figure 1 and 2 showed the enrichment of GCN5 and CAMTA2 in WT and corresponding knock-out lines, respectively (Two replications were shown here). We observed only minimal enrichment of GCN5 or CAMTA2 at the promoter regions of these six genes, and the

differences compared to the mutants were not stable and statistically significant among multiple ChIP experiments.

Figure 1 Enrichment of GCN5 by ChIP-seq in WT and *gcn5* knock-out lines. The arrows indicate the direction of transcription.

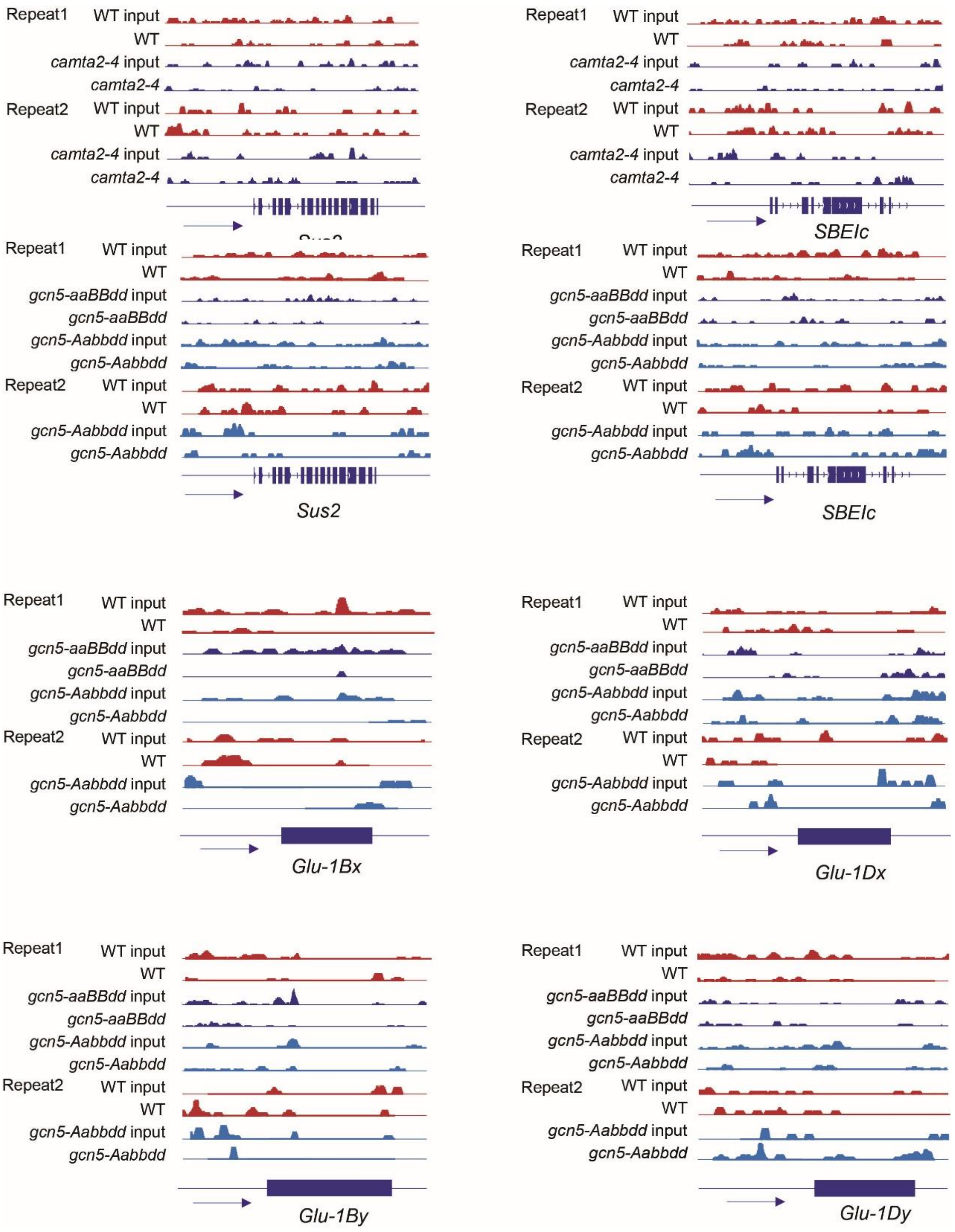

Figure 2 Enrichment of CAMTA2 by ChIP-seq in WT and *camta* knock-out line.The arrows indicate the direction of transcription.

To address this issue, we improved the ChIP–qPCR experimental design to mitigate biased enrichment. We randomly selected 15 genes evenly distributed over wheat chromosomes and designed two pairs of primers for each gene to target promoter and gene-body regions. When the ratio of IP to input enrichment exceeds 1 in the WT, it indicates the presence of GCN5 enrichment. Out of the 15 genes we studied, we found that two genes howed enrichment of GCN5 in their promoter or gene-body regions. GCN5 enrichment in one of these genes was significantly reduced in *gcn5* mutants, which suggests that only one gene was directly regulated by GCN5. However, the other 14 genes are not regulated by GCN5. We added these results in **Supplemental Figure 3D**. Furthermore, for six GCN5-targeted genes, including *Sus2*, *SBEIc*, and four *Glu* genes, we designed an additional three pairs of primers covering promoter and gene bodies. GCN5 is enriched in the promoter region but not in gene bodies. We added these results in revised **Figure 2D** and **Figure 7D**. Although we could not provide ChIP-seq results as suggested by the reviewer, our ChIP–qPCR findings confirm the specific binding of GCN5 to its targets and demonstrate a lack of enrichment in randomly selected non-target genes.

My group and co-author Dr. Xiao Jun's lab have succeeded in ChIP-seq experiments using antibodies against histone modifications. Unfortunately, we have faced significant challenges with antibodies against transcription factors and other proteins, which is also a problem in wheat research as there have been no publications on this yet. Recognizing the importance of ChIP-seq data, we are committed to refining our methodology to obtain more conclusive results. However, we also wish to publish our findings as soon as possible to highlight the novelty of GCN5 and CAMTA2. One of the reasons why GCN5 and CAMTA2 antibodies are apparently ineffective in providing a sufficient amount of immunoprecipitation is that they are not strong enough, as compared to the no-Ab negative control. Secondly, GCN5 and

CAMTA2 may exhibit low affinity for DNA, especially GCN5, leading to an insufficient pull-down of the protein–DNA complex, resulting in reduced signal strength. Thirdly, high amounts of polysaccharides and polyphenols present in wheat endosperm lead to challenges for ChIP-seq in this tissue.

2. GCN5 in Arabidopsis, yeast and mammals (DOI 10.1074/jbc.M111.329417 ; doi.org/10.1371/journal.pone.0054896 ; DOI:10.1111/J.1365-313X.2007.03264.X ; doi: 10.1093/nar/gkaa369.) it is clearly demonstrated that the primary site of acetylation by Gcn5 is H3K14. However, in the results displayed by the authors it seems that in wheat it is not the case. (i) Is it really GCN5 that is mutated or another HAT? (ii) How authors can explain that in Arabidopsis, yeast and mammals GCN5 target H3K14 and not in wheat?

Response: We thank the reviewer for this suggestion. Firstly, we made sure that only *GCN5* was mutated in *gcn5* knock-out lines. We ensured the specificity of the sgRNA used for targeting a conserved region of all three *GCN5* homoeologs and avoided off-target editing of other *HATs*. We previously generated a *GCN5* mutant using CRISPR–Cas9 in the three *GCN5* homoeologs in the c.v. Fielder background that contained homozygous mutations in the A and B homoeologs only, with the D homeolog unaffected (Zheng et al., 2021). Here, we obtained three additional genetically independent homozygous mutants including *gcn5-AABBdd*, *gcn5-AAbbdd*, *gcn5-aaBBdd* and two heterozygous lines including *gcn5-Aabbdd* and *gcn5-aaBbdd* by PCR and sequencing techniques. Figure 3A below in this letter displays the sequences of wild-type c.v. Fielder and the five *gcn5* mutants around the sgRNA binding site in the three GCN5 homoeologs. Thus, *GCN5* was indeed mutated in these lines. To analyze off-targeting, we examined the single-guide RNAs (sgRNAs) targeting *GCN5-A*, *GCN5-B*, and *GCN5-D* by BLAST to the Ensembl Plants database. We obtained eight similar sequences, but none of them contained the protospacer adjacent motif (PAM) sequence (NGG), which theoretically precludes off-target events. To further confirm off-targeting, we sequenced these eight loci in five *gcn5*

mutant lines, and we found that they were not altered. In addition, RNA-seq analysis confirmed that the expression of *GCN5* is down-regulated in two independent *gcn5* mutant lines (*gcn5-aaBBdd* and *gcn5-Aabbdd*), while no statistically significant changes were observed in the expression levels of the other 14 *HAT* genes. We added these results in **Supplemental Figure 1C**.

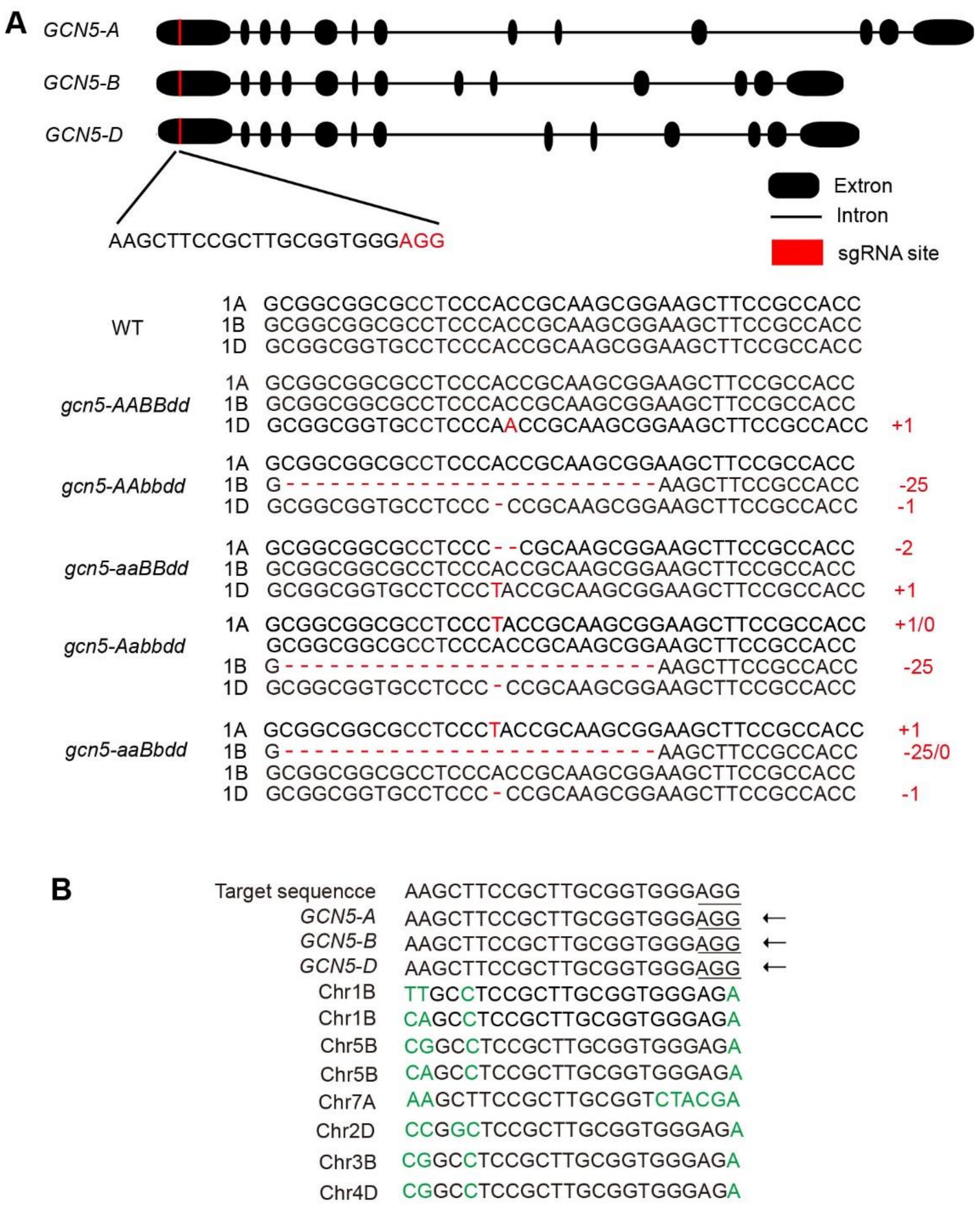

Figure 3 Sequences of wild-type and the five *gcn5* mutants

Previous studies have shown that GCN5 is responsible for acetylation of H3K9 and H3K14 in Arabidopsis, rice, wheat, maize, yeast and mammals. HAG1/GCN5 affects the expression of *PAD4* via H3K9 and/or H3K14 acetylation in wheat (Song et al., 2022). In Arabidopsis, GCN5 regulates stem cuticular wax synthesis by activating the acetylation level of H3K9/K14 in the *ECERIFERUM3* (*CER3*) promoter region (Wang et al., 2018). In this study, we saw that the overall H3K9ac levels were decreased in the *gcn5* mutant, while H3K14ac total levels remained unchanged

through western blot analysis. It is possible that H3K14 level in some loci or genes were changed, but it is still unclear. We propose two hypotheses regarding the regulation of H3K14ac by GCN5 in wheat. The first hypothesis suggests that GCN5 may regulate H3K14ac at specific gene loci, resulting in changes without affecting the global H3K14ac levels. However, we have not yet conducted any experiments to confirm this idea. The second hypothesis proposes that due to the unique nature of the endosperm tissue, GCN5 may interact with specific transcription factors in different tissues to regulate the acetylation levels of various downstream target genes differently. This would lead to variable modifications of H3K9ac and H3K14ac. We added this point in the discussion part of the revised manuscript (**Line 439–452**).

References:

Zheng M, Lin J, Liu X, Chu W, Li J, Gao Y, An K, Song W, Xin M, Yao Y, Peng H, Ni Z, Sun Q, Hu Z. Histone acetyltransferase TaHAG1 acts as a crucial regulator to strengthen salt tolerance of hexaploid wheat. **Plant Physiol.** 2021 Aug 3;186(4):1951-1969. doi: 10.1093/plphys/kiab187. PMID: 33890670; PMCID: PMC8331135.

Song N, Lin J, Liu X, Liu Z, Liu D, Chu W, Li J, Chen Y, Chang S, Yang Q, Liu X, Guo W, Xin M, Yao Y, Peng H, Ni Z, Xie C, Sun Q, Hu Z. Histone acetyltransferase TaHAG1 interacts with TaPLATZ5 to activate TaPAD4 expression and positively contributes to powdery mildew resistance in wheat. **New Phytol.** 2022 Oct;236(2):590-607.

Wang T, Xing J, Liu X, Yao Y, Hu Z, Peng H, Xin M, Zhou DX, Zhang Y, Ni Z. GCN5 contributes to stem cuticular wax biosynthesis by histone acetylation of CER3 in Arabidopsis. **J. Exp. Bot**. 2018 May 25;69(12):2911-2922. doi: 10.1093/jxb/ery077. PMID: 29506042; PMCID: PMC5972625.

3. As for GCN5, a ChIP-seq for CAMTA2 is mandatory.

Response: Please see the response to comment #1. To further investigate the binding specificity of CAMTA2, we also randomly selected 13 genes for ChIP–qPCR analysis.

And designed two pairs of primers for each gene to target promoter and gene-body regions When the ratio of IP to input enrichment exceeds 1 in the WT, it is indicative of CAMTA2 enrichment. Among the 13 genes, we found that no gene showed enrichment of CAMTA2 in its promoter region or gene-body region. We added these results in **Supplemental Figure 9B**. Furthermore, for six CAMTA2-targeted genes including *Sus2*, *SBEIc* and four *Glu* genes, we designed additional three pairs of primers, totaling four pairs, covering promoter and gene-body regions of these genes (revised **Figure 5B and 7H**). The observation indicated that CAMTA2 enriched in promoter region around its binding motif but not other regions.

4. Authors showed in their model that GCN5 and CAMTA2 binds chromatin at the same position. To prove that authors need to perform a ChIPre ChIP experiment.

Response: We have tried our best to perform Re-ChIP experiments in wheat endosperm, but have been unable to obtain positive results. As you may know, wheat is a complex hexaploid (AABBDD, 2n = 6x = 42), with a genome size of approximately 15 Gb, more than 90% of which is composed of non-coding regions that harbor mostly regulatory elements. This high degree of repetitiveness and complexity amplifies the difficulty of identifying specific DNA regions within the genomic background. The endosperm is a specialized tissue that requires careful handling and processing to preserve the integrity of chromatin and protein–DNA interactions. The isolation of high-quality chromatin from wheat endosperm is technically demanding.

Reviewer #2 (Comments for the Author):

A major breeding goal is to boost grain weight in wheat. Of them, starch acts as a predominant role in determining grain weight. However, the improvement of starch content is a major challenge for modern agriculture due to the limited understanding of the molecular regulatory network by which starch is synthesized and accumulated in developing endosperm. In this manuscript, the authors generated a GCN5 mutant using the CRISPR/Cas9 strategy, and focused on the grain phenotypic defect of the

gcn5 mutant. The further experiment revealed that the gcn5 mutation differentially affects the expression of genes associated with starch and storage protein biosynthesis through directing binding to their promoters. More strikingly, they also identified the transcription factor CAMTA2 as the interacting partner of GCN5 to cooperatively affect starch but not storage proteins. The topic of this manuscript is interesting for readers. Overall, the experiments in this manuscript are well conducted, and the data is well present. However, the cellular and genetic data are relatively weak. Below, please see my suggestions for revising or improving the manuscript:

Major comments:

1. To understand the cellular mechanism of GCN5 and CAMTA2 to regulate wheat grain size and weight, the cellular section analyses of wheat grain need to be done. Besides, from the RNA sequencing data, the affected cell expansion genes, cell division genes and hormone related genes need to be selected for RT-qPCR to analyze the molecular mechanism of GCN5 and CAMTA2 to regulate wheat grain size and weight.

Response: We thank the reviewer for this suggestion. We have now measured the cell size and cell number within the same area of 15-DAP endosperm from WT, *gcn5* and *camta2* mutants. There is no statistically significant alteration in either cell size or cell number in *gcn5* and *camta2* mutants compared to WT (**Supplemental Figures S2F–H and S7D–F**). This analysis was added in the Results (**Lines 171-174, Lines 290-291**). These results suggest that GCN5 and CAMTA2 might regulate grain size and weight by influencing the accumulation of key storage components, such as starch and storage protein.

Our RNA-seq analysis identified down-regulation of genes in the *gcn5* mutant associated with brassinosteroid (BR) biosynthesis. The expression levels of two down-regulated genes including TraesCS4A02G078000, which encodes a Cytochrome P450 90B2 and is an ortholog of rice *DWARF4*/*CYP90B2*, and TraesCS2A02G013500, an ortholog of rice *brd1*/*DWARF* were confirmed using RT–qPCR (**Supplemental Figure S3B**). We have included the results in the revised

manuscript (**Lines 195–203**).

2. The authors claimed that GCN5 and CAMTA2 work cooperatively to regulate storage substance accumulation. The only data to support this proposition is the phenotypic data of single mutants. The reviewer suggests the authors to generate a double mutant to enhance their conclusion. If possible, complementation test should also be supplemented for knock-out mutants mentioned in this manuscript.

Response: We have successfully carried out a genetic cross between the *gcn5* (*gan5-Aabbdd*) and *camta2* (*camta2-4*) mutant and expect to harvest the F_1 generation seeds in May 2024. Following this, we intend to propagate the F_1 seeds within a greenhouse environment, wherein they will undergo self-pollination to yield F_2 seeds. Subsequently, the F_2 seeds will be planted, and a segregation analysis will identify and isolate the double mutants. These double mutants will then be transplanted to the field for phenotypic assessment. We are confident that the detailed phenotypic analysis of the double mutants will explain the cooperative regulatory interactions between the *gcn5* and *camta2* genes to regulate storage substance accumulation. However, we cannot predict a precise timeline for the confirmation of these mechanisms. Furthermore, we have successfully constructed complementation vectors. However, the outcomes of the complementation assays remain pending due to time limitations. Considering the importance of this suggestion, we added these descriptions to the Discussion of the revised manuscript (**Lines 512–518**).

3. The reviewer noticed that numerous genes associated with storage and starch proteins are also seriously down-regulated in the *gcn5* or *camta2* mutants. The possible explanations should be discussed.

Response: Thank you for raising this point. We propose that GCN5 or CAMTA2 may regulate these down-regulated genes in two ways and further described these models in the Results and Discussion sections: **(1)** GCN5 or CAMTA2 directly regulate the expression of some genes. For instance, *gcn5* mutations lead to a reduction in H3K9ac levels at the promoter regions of *Sus2*, *SBEIc* and *Glu*, resulting in decreased

expression. CAMTA2 can directly bind to the CGCG elements in their promoters and activate their expression. Mutation of CAMTA reduces the expression of *Sus2*, *SBEIc* and *Glu* genes. **(2)** GCN5 or CAMTA2 indirectly affects the expression of some genes. For example, in the *gcn5* mutant, the expression of *NF-YB1*, a transcription factor that positively regulates the expression of starch-biosynthesis genes (Liu et al., 2022), is reduced (**Supplemental Figure S3C**). GCN5 may indirectly influence the expression of starch and seed-storage-protein genes by regulating the expression of *NF-YB1* (**Lines 210–213 and 434–438**). In *camta2* mutants, PBF/LYS3, which positively regulates starch accumulation (Orman-Ligeza et al., 2020), and SPA, a transcription factor that positively regulates the expression of seed-storage-protein genes (Albani et al., 1997; Ravel et al., 2014) exhibit down-regulated expression (**Supplemental Figure S8B and 12B**). Both genes have CGCG elements in their promoter regions, suggesting that CAMTA2 potentially modulates genes associated with storage and starch proteins by directly binding to *PBF/LYS*3 (**Lines 304–307 and 468–470**) and *SPA* promoters (**Lines 401–405 and 485–488**). These descriptions were added to the Results and Discussion section of the revised manuscript.

References:

Liu G, Zhang R, Li S, Ullah R, Yang F, Wang Z, Guo W, You M, Li B, Xie C, Wang L, Liu J, Ni Z, Sun Q, Liang R. TaMADS29 interacts with TaNF-YB1 to synergistically regulate early grain development in bread wheat. **Sci. China Life Sci.** 2023 Jul;66(7):1647-1664. doi: 10.1007/s11427-022-2286-0.

Orman-Ligeza B, Borrill P, Chia T, Chirico M, Doležel J, Drea S, Karafiátová M, Schatlowski N, Solomon CU, Steuernagel B, Wulff BBH, Uauy C, Trafford K. LYS3 encodes a prolamin-box-binding transcription factor that controls embryo growth in barley and wheat. **J. Cereal Sci.** 2020 May; 93: 102965.

Ravel C, Fiquet S, Boudet J, Dardevet M, Vincent J, Merlino M, Michard R, Martre P (2014) Conserved cis-regulatory modules in promoters of genes encoding wheat high-molecular-weight glutenin subunits. **Front. Plant Sci.** 5: 62

4. Given that GCN5 has been widely reported in plants, the novelty of this manuscript should be further enhanced through revising the text to highlight the light spot.

Response: We thank the reviewer for this recommendation. Although GCN5 has been well-characterized in plants and animals, its function and molecular mechanisms in wheat grain development have barely been explored. Guo et al. suggest a potential interaction between GCN5 and the GAMYB transcription factor to regulate *Glu*. We have discovered that GCN5 not only regulates the accumulation of storage proteins but also participates in regulating starch accumulation, thereby affecting grain size and weight. Furthermore, we found that GCN5 modulates gene expression by controlling the H3K9ac levels in the promoters of starch-biosynthesis and glutenin genes, thereby expanding the role of histone acetylation in the regulation of grain development. Few genes are known to be involved in the concomitant regulation of wheat seed-storage proteins and starch accumulation and this provides insights into this. Additionally, we identified an interaction between wheat GCN5 and the calmodulin-binding transcription factor CAMTA2, which regulates grain size and weight, offering valuable genetic resources for high-yield wheat breeding. These descriptions were added to the Introduction section of the revised manuscript (**Lines 136–138**).

Minor comments:

1. The meanings of the sentence "gcn5 knocks have down-regulated Glu transcripts levels encoding high molecular weight (HMW) glutenin and to lower levels of HMW glutenin protein, gluten contents and SDS sedimentation volume" is not well clear, please revise this sentence to depict clearly its meanings.

Response: We have revised this sentence following the reviewer's suggestion (**Lines 28–31**).

2. In Figure 2D, the ratio of different H3 acetylation to H3 proteins need be calculated to well display the different H3 acetylation change pattern.

Response: We thank the reviewer for this suggestion. We have calculated the ratio of

different H3 acetylation to H3 proteins following the reviewer's suggestion in **Figure 2E**.

3. From Supplemental Figure 1c, this claim "No visible phenotypic differences were detected in gcn5 mutant mature plants relative to wild type, including plant height, tiller number and spike number" is not well supported. The Authors need to collect the statistical data of plant height, tiller number and spike number to support their claim.

Response: We did not examine tiller numbers and spike numbers as incorrectly mentioned in the original manuscript. We quantified and statistically analyzed plant height, panicle length, spikelet number and grains per panicle (**Supplemental Figure S2A–E**) and revised this sentence (**Line 158**).

4. In Figure 3D, E, the font in the right boxes is too small to see, please use bigger fonts.

Response: Thank you. We have enlarged the fonts.

5. Seed-storage proteins genes were down-regulated in *camta2* mutants, why the seed-storage proteins are unaffected in camta2 mutants? What is the possible mechanism?

Response: The possible mechanisms might be associate with translational regulation. Adjustments in translation efficiency, such as optimization of the initiation and elongation processes, could maintain stable protein synthesis despite transcriptional downregulation (Verma et al., 2019). Moreover, enhanced protein stability, achieved through post-translational modifications like phosphorylation, also contributes to maintaining a constant protein abundance amidst transcriptional fluctuations (Hu et al., 2023). Other components involved in translational regulation of SSPs genes were regulated by CAMTA2. We added this point in discussion part (**Lines 492–499**).

6. English should be improved by a professional editor. In addition, there are some typos that should be checked throughout the manuscript.

Response: Thank you. We have revised the manuscript with assistance from a professional editor.

We once again express our appreciation for the reviewer's comments and trust that our efforts have addressed them all. Thank you for reconsidering this manuscript.

On behalf of all authors, sincerely yours,

Professor Yingyin Yao, Ph.D.
Department of Plant Genetics and Breeding,
China Agricultural University
NO.2 Yuanmingyuan Xi Road, Haidian District, Beijing
Office number: 86-10-62732304
Email: yingyin@cau.edu.cn

Prof. Yingyin Yao
China Agricultural University
Plant genetics and breeding
Beijing 100193
China

Dear Dr. Yao:

We have received reviews of your manuscript entitled "Wheat grain weight is regulated by the histone acetyltransferase GCN5 interacting with the CAMTA2 transcription factor." As you can see, while one review was satisfied, the other raised several additional critical concerns. In light of these comments, we decided to offer you another chance to respond and revise the manuscript. Please fully address the concerns either with additional experiments and/or discussion. I should stress that the final decision is pending on revision and re-review.

Please contact us if there are ambiguous comments or if you wish to discuss the revision.

Given the nature of the comments, we are offering you 60 days from when we have issued this decision to complete the revision. If a revision is not returned within this time frame, and if you have not been granted an extension, we will withdraw the manuscript, which will leave you free to submit the work elsewhere. If you need an extension, we encourage you to contact us at any point before the 60 days have passed.

When you are ready to submit the revised version, please upload a highlighted copy that indicates all changes made in response to the editor and reviewer recommendations. Include an itemized list of all changes made in response to each of the reviewer's suggestions in the "Response to Reviewers" section; please note that reviewers do not have access to your cover letter, nor was this decision letter shared with them.

Thank you for the privilege of reviewing your work. We look forward to receiving your revised manuscript.

Sincerely,

The Plant Cell Editorial Board

Please note the following:

-The Plant Cell now requires authors to complete and submit an author revisions checklist upon submission of a revised manuscript. The aim of the checklist is to aid authors in preparing a high-quality manuscript, facilitate the review and assessment of revised manuscripts, and help to ensure that journal standards are maintained across the board. If your manuscript is accepted, the completed checklist will be published as supplemental material attached to the article online. Please download a copy of the checklist (pdf fillable form) at this link, for submission with your revised manuscript: https://tpc.msubmit.net/html/Author_Revisions_Checklist.pdf.

-Supplemental materials should be restricted to large datasets and tables, presentation of replicates, and validation of reagents, methods, or genotypes. Any data that are used to support the major claims must be in the main manuscript. Supplemental figure legends must indicate what figure in the main manuscript is supported by the supplemental data presented. Please justify how each of the supplemental figures meet the criteria.

-Sampling methods and nature of "biological replicates" should be described precisely (i.e. different plants, parts of plants, pooled tissue, independent pools of tissue, sampled at different times, etc), along with a clear description of and rationale for any statistical analyses conducted. The reader should know exactly what was sampled; what forms the basis of the calculation of any means and statistical parameters reported. This is also necessary to ensure that proper statistical analysis was conducted.

-Want to add this revision deadline to your calendar? Click below!

-- Reviewer comments:

Reviewer #1 (Comments for the Author):

In their response to my first comment regarding the ChIP-seq, the authors argue that it is challenging, which I understand. However, in my view, the results of the ChIP-qPCR are not convincing enough. The fold enrichments for GCN5 ChIP are low (Figure 2), no controls for the specificity of the antibodies are presented, and there is no IgG control.
Additionally, the authors claim that their results "collectively indicate that GCN5 contributes to the regulation of Sus2 and SBEIc expression by mediating H3K9ac levels in their promoters." However, in my view, the fact that a gene downregulated displays a reduction of H3K9ac is expected and does not prove that the mutated gene, which the authors named GCN5, is responsible for this effect.
As I mentioned in my first review, GCN5 is well-known to acetylate H3K14ac. However, according to the authors' data, this does not seem to be the case in wheat. This discrepancy is very intriguing and a major point for me. How can an enzyme conserved in yeast, animals, and plants have the same lysine target in all tested organisms (Arabidopsis, yeast, Drosophila, human, etc.) but not in wheat? Either what the authors named GCN5 is not actually GCN5 but another HAT with a bromodomain, or if it is indeed GCN5, it is important to determine why it affects H3K9ac more than H3K14ac.
In their model, GCN5 seems to be able to bind chromatin thanks to its capacity to interact with transcription factors, but what about its bromodomain?
In my view, the work is interesting, but the molecular mechanism behind the phenotype is not clear at all.

Reviewer #2 (Comments for the Author):

The authors have adequately addressed most of my concerns, in addition to the genetic experiments due to time constraints. I appreciated their efforts, and have no further comments. This revised version of manuscript is acceptable for publication in the Plant Cell.

Dear Editor and Reviewers,

We wish to thank you all for your time, assistance, and suggestions for the improvement of our manuscript. We have revised the manuscript ' **Wheat grain weight is regulated by the histone acetyltransferase GCN5 interacting with the CAMTA2 transcription factor**' (Manuscript ID TPC2023-RA-01206R1), wherein we have done our best to robustly address all the reviewers' concerns and suggestions.

Below we provide point-by-point responses (in dark brown) to the reviewers' comments. The indicated line numbers refer to the revised manuscript wherein revisions are highlighted by track changes.

Reviewer #1 (Comments for the Author):

In their response to my first comment regarding the ChIP-seq, the authors argue that it is challenging, which I understand. However, in my view, the results of the ChIP-qPCR are not convincing enough. The fold enrichments for GCN5 ChIP are low (Figure 2), no controls for the specificity of the antibodies are presented, and there is no IgG control.

Response: In the revised manuscript, we re-conducted all ChIP-qPCR experiments using antibodies against anti-GCN5, CAMTA2, H3K14ac, and H3K9ac as suggested by the reviewer, following the experimental methods from "*The Plant Cell*" (Li et al., 2015; Chun et al., 2023; Wang et al., 2024). We made the following modifications to the ChIP-PCR as suggested by the reviewer: 1) We used anti-IgG as a control instead of the previous use of mock (no antibody added) as a negative control. 2) We refined the method for calculating the ChIP values. Previously, the ChIP values were normalized to the input sample. In the revised manuscript, the ChIP values were normalized to the input sample, and the enrichment was calculated based on the relative enrichment of immunoprecipitation with anti-GCN5, CAMTA2, H3K14ac, and H3K9ac antibodies compared to that with anti-IgG immunoprecipitation. The

results we obtained are consistent with our previous experimental findings. The new data are shown in Figure 2C, Figure 2D, Figure 2H, Figure 5B, Figure 5E, Figure 5F, Figure 7D, Figure 7E, Figure 7I, Figure 7J, Supplemental Figure S3D, and Supplemental Figure S9B. We have also made revisions to the Materials and Methods section in the revised manuscript (Lines 605–608).

We understand the concern about the specificity of the GCN5 and CAMTA2 antibodies. At the moment, we don't have appropriate positive controls specifically for these antibodies, as our knowledge of GCN5 and CAMTA2 target genes in wheat endosperm is limited. However, we did use randomly selected genes as negative controls. Our ChIP-qPCR experiment results revealed that only one of the 15 randomly selected genes, which were evenly distributed across the wheat chromosomes, is directly regulated by GCN5 (refer to Supplemental Figure S3D). None of the 13 randomly selected genes were found to be target genes of CAMTA2 (refer to Supplemental Figure S9B). These results clearly indicate the specific binding of GCN5 and CAMTA2 to their targets.

References:

1. Li C, Qiao Z, Qi W, Wang Q, Yuan Y, Yang X, Tang Y, Mei B, Lv Y, Zhao H, Xiao H, Song R. Genome-wide characterization of *cis*-acting DNA targets reveals the transcriptional regulatory framework of *opaque2* in maize. Plant Cell. 2015.

2. Chun Y, Fang J, Savelieva EM, Lomin SN, Shang J, Sun Y, Zhao J, Kumar A, Yuan S, Yao X, Liu CM, Arkhipov DV, Romanov GA, Li X. The cytokinin receptor OHK4/OsHK4 regulates inflorescence architecture in rice via an IDEAL PLANT ARCHITECTURE1/WEALTHY FARMER'S PANICLE-mediated positive feedback circuit. Plant Cell. 2023.

3. Wang YG, Zhang YM, Wang YH, Zhang K, Ma J, Hang JX, Su YT, Tan SS, Liu H, Xiong AS, Xu ZS. The Y locus encodes a REPRESSOR OF PHOTOSYNTHETIC GENES protein that represses carotenoid biosynthesis via interaction with APRR2 in carrot. Plant Cell. 2024.

Additionally, the authors claim that their results "collectively indicate that GCN5 contributes to the regulation of Sus2 and SBEIc expression by mediating H3K9ac levels in their promoters." However, in my view, the fact that a gene downregulated displays a reduction of H3K9ac is expected and does not prove that the mutated gene, which the authors named GCN5, is responsible for this effect.

Response: We appreciate the reviewer's suggestion. We agree that our conclusion was not precise enough. In the revised manuscript, we have revised this sentence as follows: The down-regulation of *Sus2* and *SBEIc* expression is associated with decreased levels of H3K9ac and H3K14ac in their promoters (Lines 231–233).

As I mentioned in my first review, GCN5 is well-known to acetylate H3K14ac. However, according to the authors' data, this does not seem to be the case in wheat. This discrepancy is very intriguing and a major point for me. How can an enzyme conserved in yeast, animals, and plants have the same lysine target in all tested organisms (Arabidopsis, yeast, Drosophila, human, etc.) but not in wheat? Either what the authors named GCN5 is not actually GCN5 but another HAT with a bromodomain, or if it is indeed GCN5, it is important to determine why it affects H3K9ac more than H3K14ac.

Response: We appreciate the reviewer's suggestion. We conducted ChIP-qPCR using Anti-H3K14ac to examine whether GCN5 affects target gene expression by controlling H3K14ac. The ChIP-qPCR results showed that the enrichment of H3K14ac in the promoters of *Sus2*, *SBEIc*, *Glu-1Bx*, *Glu-1By*, *Glu-1Dx*, and *Glu-1Dy* was decreased in *gcn5* mutants (Figure 2I, 7F). Additionally, the enrichment of H3K14ac in the promoters of *Sus2* and *SBEIc* was also reduced in *camta2* mutant (Figure 5G). These findings suggest that the H3K14ac levels in specific loci or genes were altered in *gcn5* mutants without impacting the global H3K14ac levels. We have included these results in the revised manuscript's Results (Lines 228–231, Lines 319-320 and Lines 365-367) and Discussion (Lines 424–426) sections.

In their model, GCN5 seems to be able to bind chromatin thanks to its capacity to interact with transcription factors, but what about its bromodomain?

Response: We thank the reviewer for the suggestion. We apologize for overlooking the function of the bromodomain of GCN5 in our previous model, where we focused solely on the recruitment of GCN5 by transcription factors to regulate downstream target genes. Bromodomains specifically bind to acetylated lysine residues on histone tails, and play an important role in anchoring the complexes of which they are a part to acetylated chromatin (Hanquier et al., 2020, Cieniewicz et al., 2014). Consequently, in the revised model, we depict GCN5 in proximity to chromatin to indicate the bromodomain function (see Figure 8).

References:

1. Hanquier J, Gimeno T, Jeffers V, Sullivan WJ Jr. Evaluating the GCN5b bromodomain as a novel therapeutic target against the parasite Toxoplasma gondii. Exp Parasitol. 2020.

2. Cieniewicz AM, Moreland L, Ringel AE, Mackintosh SG, Raman A, Gilbert TM, Wolberger C, Tackett AJ, Taverna SD. The bromodomain of Gcn5 regulates site specificity of lysine acetylation on histone H3. Mol Cell Proteomics. 2014.

Reviewer #2 (Comments for the Author):

The authors have adequately addressed most of my concerns, in addition to the genetic experiments due to time constraints. I appreciated their efforts, and have no further comments. This revised version of manuscript is acceptable for publication in the Plant Cell.

Response: We thank the reviewer for this suggestion. We have completed a successful genetic cross between the *gcn5* (*gan5-Aabbdd*) and *camta2* (*camta2-4*) mutants and harvested the F_1 generation seeds in May 2024. At present, the F_1 seeds have been planted in greenhouse and will undergo self-pollination to generate F_2 seeds.

We once again express our appreciation for the reviewer's comments and trust that our efforts have addressed them all.

Thank you for reconsidering this manuscript.

On behalf of all authors, sincerely yours,

Professor Yingyin Yao, Ph.D.

Department of Plant Genetics and Breeding,

China Agricultural University

NO.2 Yuanmingyuan Xi Road, Haidian District, Beijing

Office number: 86-10-62732304

Email: yingyin@cau.edu.cn

Dear Dr. Yingyin Yao:

We are pleased to inform you that your paper entitled "Wheat grain weight is regulated by the histone acetyltransferase GCN5 interacting with the CAMTA2 transcription factor" has been accepted for publication in The Plant Cell, pending a final editorial review by a science editor. At this stage, your manuscript will be evaluated by a science editor with respect to its presentation of scientific content, compliance with journal policies, and presentation for a broad readership. The Plant Cell has contracted with Plant Editors (planteditors.com) to provide this service to our authors, and you will soon receive additional information on this process.

Please note that each author needs to link their ORCID identifier to their account in the system before your manuscript can be published. If any authors do not have an ORCID linked to their account, they will receive a message with a link to complete this task. Please ensure that ALL of your coauthors have completed this task as soon as possible.

LICENSE INFORMATION Shortly after your paper is sent to production, you will receive an email from our publisher's author support team, **SciPris**, with information on article processing charges, as well as the types of Open Access license you would be able to purchase. The accepted version of your manuscript will not appear on our Advance Articles page until the license is selected and signed, so please look out for an email from SciPris. For more information see the Article Publication Fees section of our author guidelines: https://academic.oup.com/plcell/pages/General-Instructions

The Plant Cell and The Arabidopsis Information Resource (TAIR) are collaborating to collect functional annotation data about Arabidopsis genes from authors. This includes information about the gene's molecular function (e.g., kinase activity, ATP synthetase activity), the biological process/es it is involved in (e.g., endosperm development, threonine biosynthesis), its subcellular location (e.g., nucleus, ER), anatomical or developmental expression pattern (e.g., leaf, ovule, flower stage 10, seedling stage), or its partner in a protein-protein interaction (e.g., AT1G01010 interacts with AT1G01020).

If your paper contains results falling into one or more of these categories for Arabidopsis genes, we request that you now submit these data for inclusion in TAIR by filling in the form provided at the following URL: https://www.arabidopsis.org/doc/submit/functional_annotation/123. If you need further clarification on what types of data can be submitted please contact curator@arabidopsis.org.

Finally, we encourage your submission of artwork for the journal cover. If there is an image or illustration related to your paper that you would like to have considered, please email it in .pdf or .tif format to clowe@aspb.org. An image title, brief, 2-3 sentence description of the image, and the name of the person credited for the image are also encouraged. If your image is selected to appear on the online cover of the journal, you will be contacted with the scheduled issue date and further information.

We look forward to seeing your paper published.

Sincerely,

The Plant Cell Editorial Board
